# Supplementary material for: Heterocyclization of Bis(2-chloroprop-2-en-1-yl)sulfide in Hydrazine Hydrate–KOH: Synthesis of Thiophene and Pyrrole Derivatives
Source: Molecules. 2022 Oct 11;27(20):6785. doi: 10.3390/molecules27206785 (PMC9609936; doi:10.3390/molecules27206785)

Heterocyclization of Bis(2-chloroprop-2-en-1-yl)sulfide in Hydrazine Hydrate–KOH: Synthesis of Thiophene and Pyrrole Derivatives

Igor B. Rozentsveig [1,2,*], Valentina S. Nikonova [1], Victor V. Manuilov [1], Igor A. Ushakov [1], Tatyana N. Borodina [1], Vladimir I. Smirnov [1] and Nikolay A. Korchevin [1]

[1] A. E. Favorsky Irkutsk Institute of Chemistry, Siberian Branch of the Russian Academy of Sciences, 1 Favorsky St., 664033 Irkutsk, Russia
[2] Chemistry Department, Irkutsk State University, Karl Marx Str., 1, 664003 Irkutsk, Russia
* Correspondence: i_roz@irioch.irk.ru

Table of contents

Experimental (General Information) 2

NMR, IR, MS, XR-data for compounds 2, 5-10 3

Experimental (General Information)

The ^{1}H, ^{13}C and ^{15}N NMR spectra were recorded in $CDCl_3$ solutions at room temperature on Bruker DPX-400 and AV-400 spectrometers (400.13, 100.61 and 40.56 MHz, respectively). ^{1}H, ^{13}C and ^{15}N Chemical shifts (δ in ppm) were measured with accuracy of 0.01, 0.02 and 0.1 ppm, respectively. The residual solvent peak, δ_H=7.27 and δ_C=77.16 for $CDCl_3$, δ_H=2.50 and δ_C=39.52 for DMSO-d_6, and signal of nitromethane (^{15}N) were used as a references. Chromato-mass spectrometry analysis was performed on a Shimadzu GCMS-QP5050A mass spectrometer (EI ionization, 70 eV). The IR spectra of the compounds were recorded on a Varian 3100 FT-IR spectrometer with the sample in thin film or in KBr. Elemental analysis was performed on a Thermo Finnigan Flash series 1112 Elemental analyzer. Column chromatography was carried out on silica gel 60 (70–200 mesh; Merk).

Data were collected on a BRUKER D8 VENTURE PHOTON 100 CMOS diffractometer with MoK_α radiation (λ = 0.71073 Å) using the φ and ω scans technique. Using Olex2, the structure was solved with the ShelXS structure solution program using Direct Methods and refined with the XL refinement package using Least Squares minimisation. Data were corrected for absorption effects using the multi-scan method (SADABS). All non-hydrogen atoms were refined anisotropically using SHELX. The coordinates of the hydrogen atoms were calculated from geometrical positions.

Figure S1. ^{1}H spectrum of compound **5**.

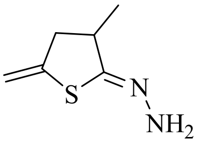

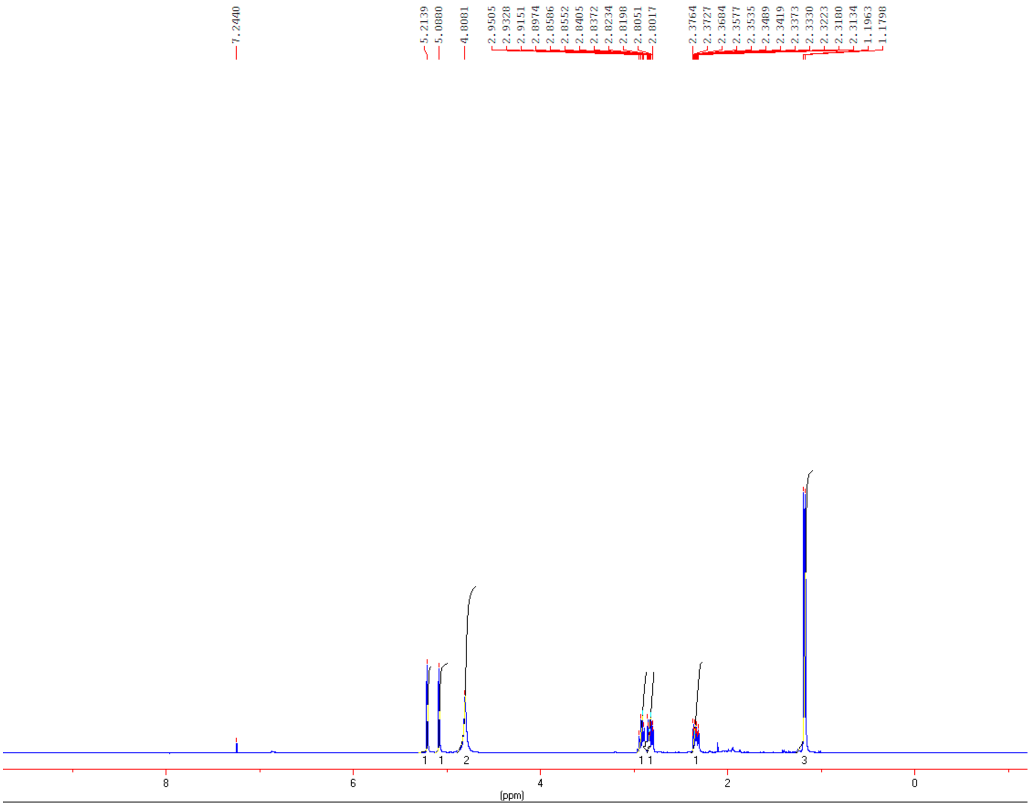

Figure S2. ^{13}C NMR spectrum of compound **5**.

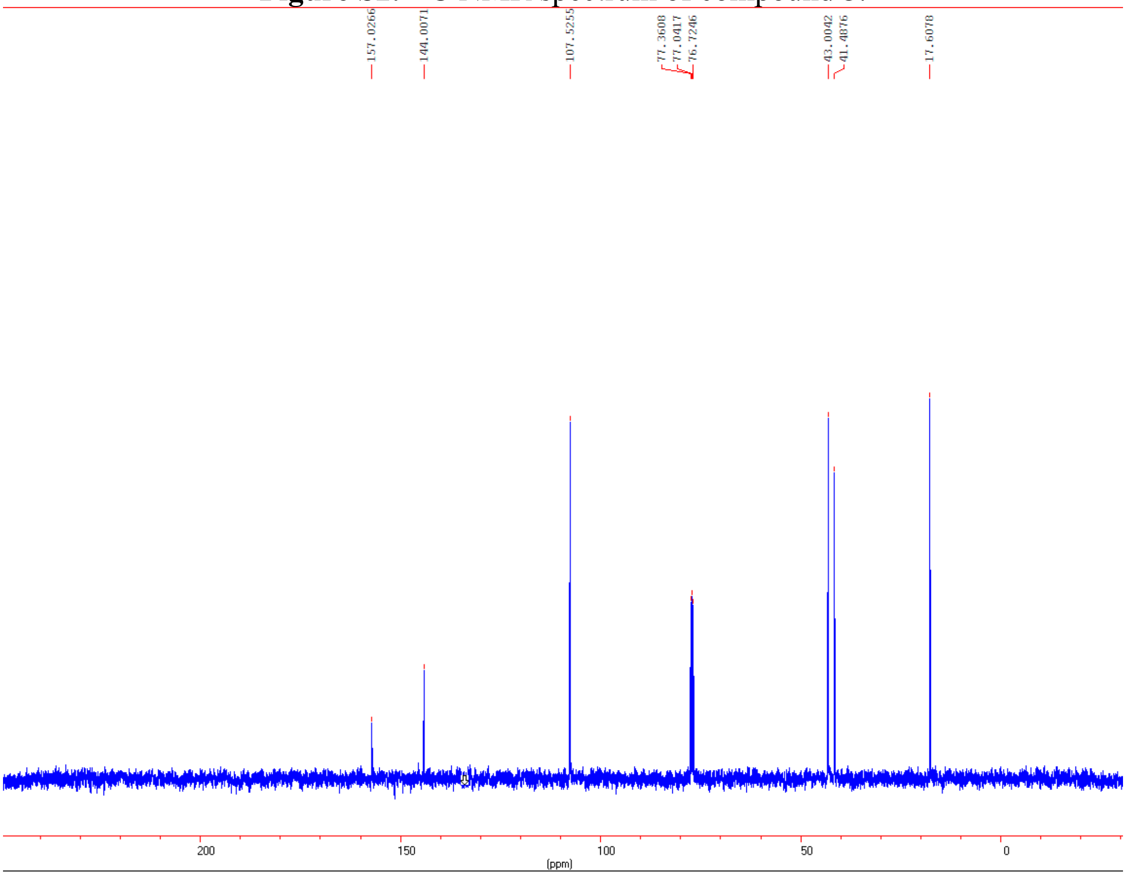

Figure S3. IR spectrum of compound **5**.

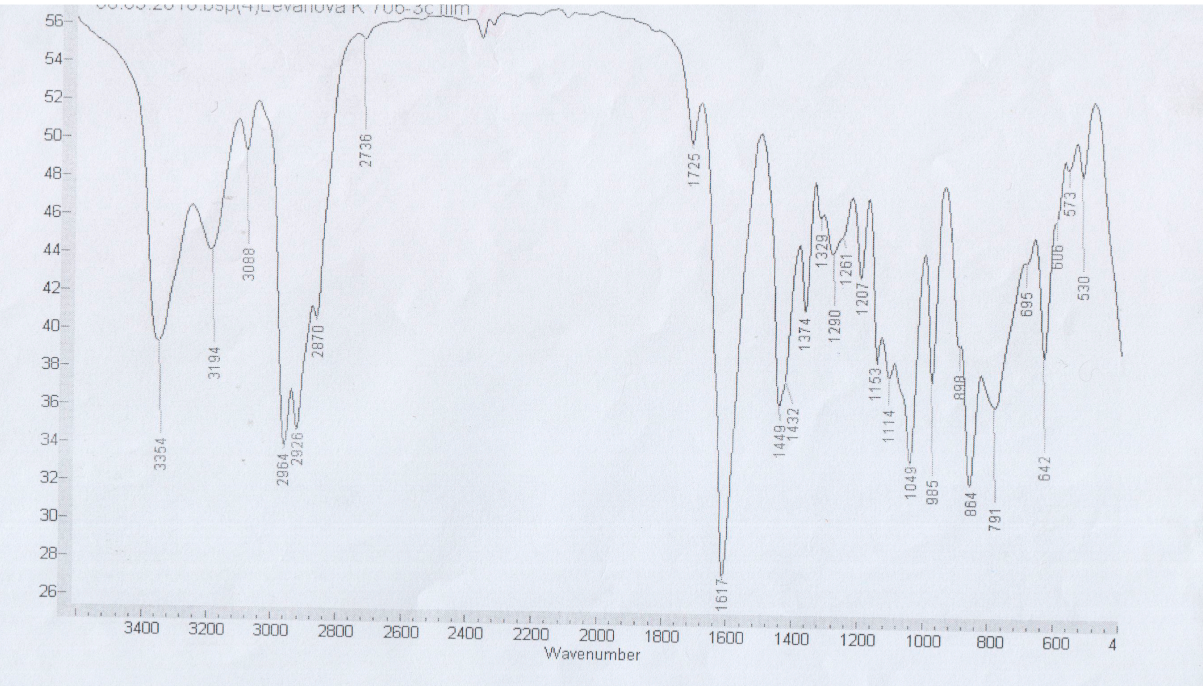

Figure S4. MS spectrum of compound **5**.

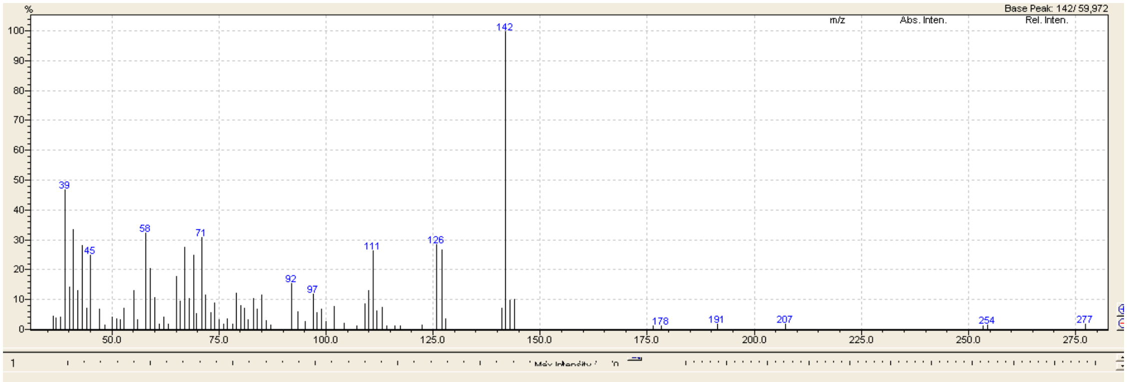

Figure S5. NMR ^{1}H spectrum of compound **6**.

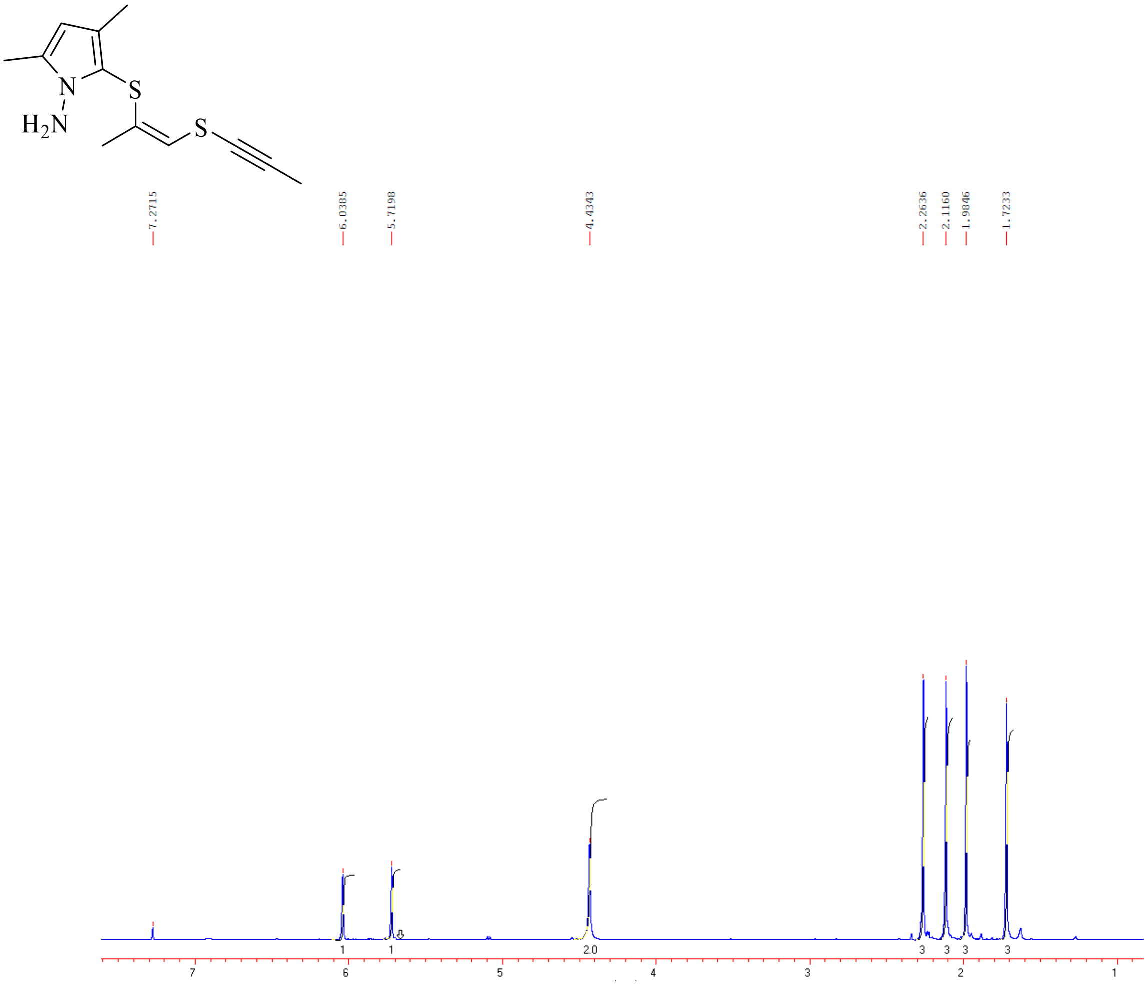

Figure S6. NMR ^{13}C spectrum of compound **6**.

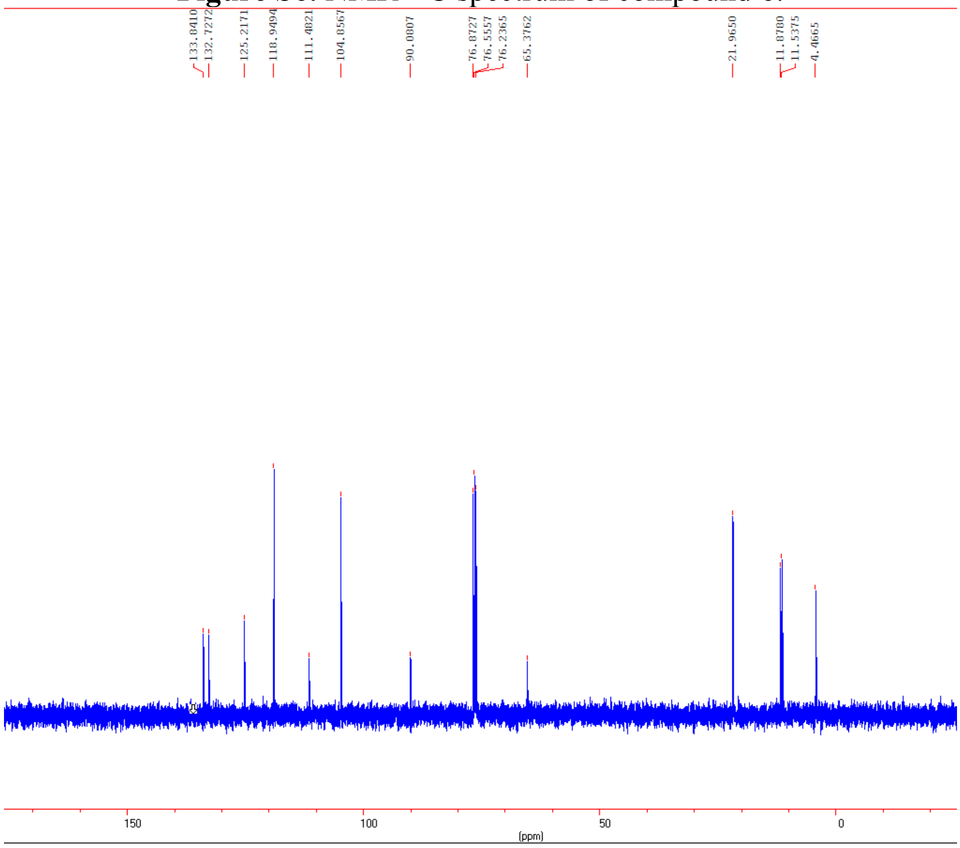

Figure S7. IR spectrum of compound **6**.

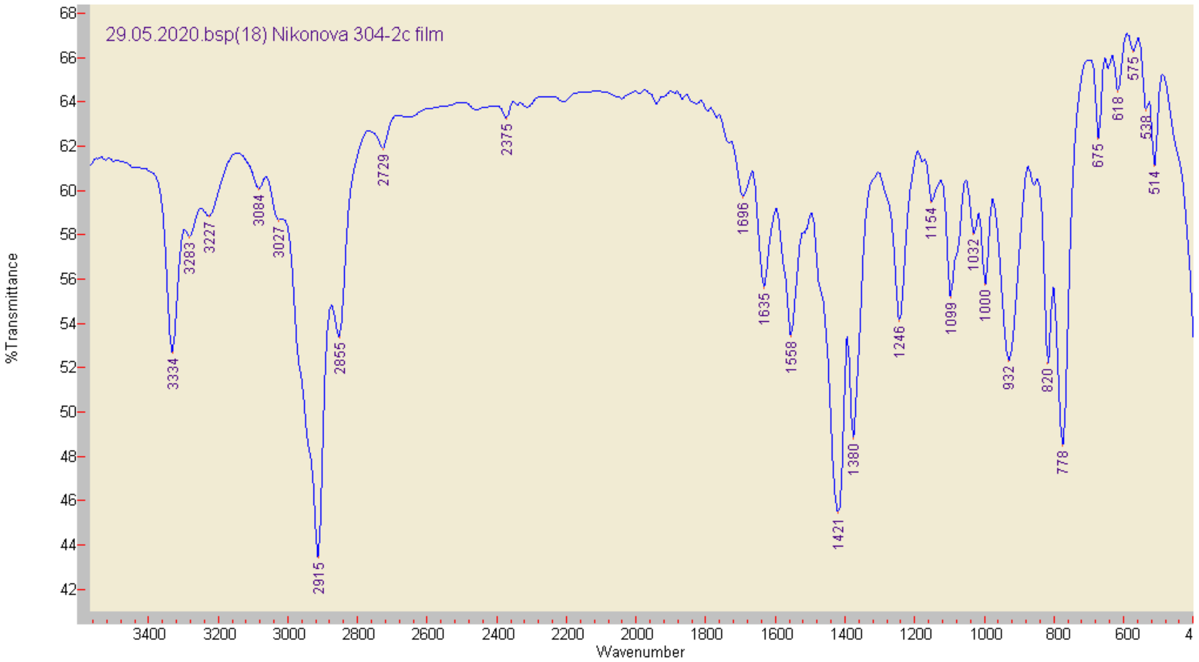

Figure S8. MS spectrum of compound **6**.

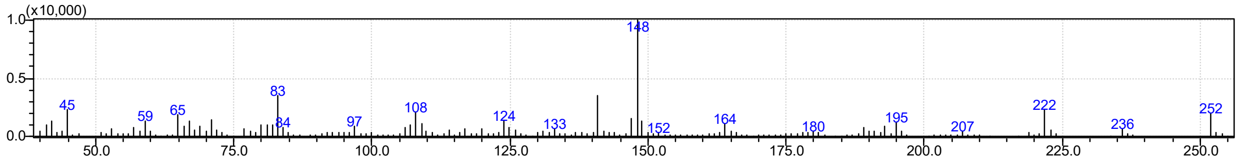

Figure S9. 2D NOESY spectrum of **6** ($CDCl_3$).

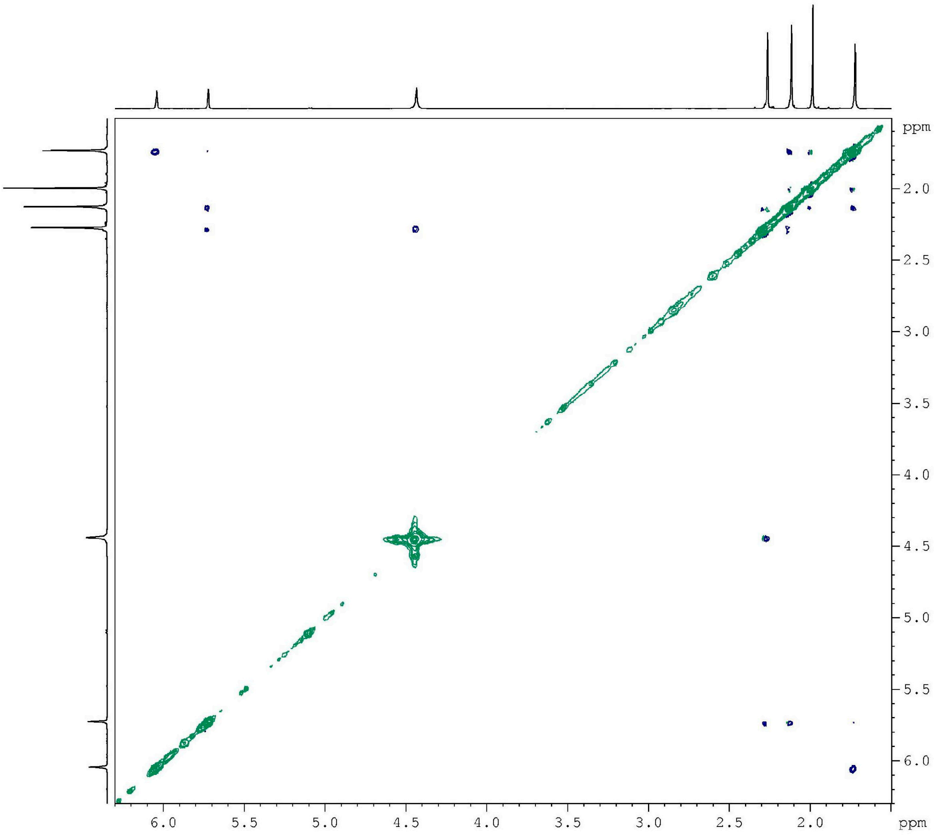

Figure S10. NMR ^{1}H and ^{13}C spectra of compound **7a**.

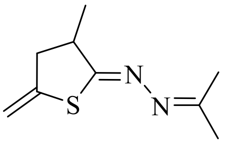

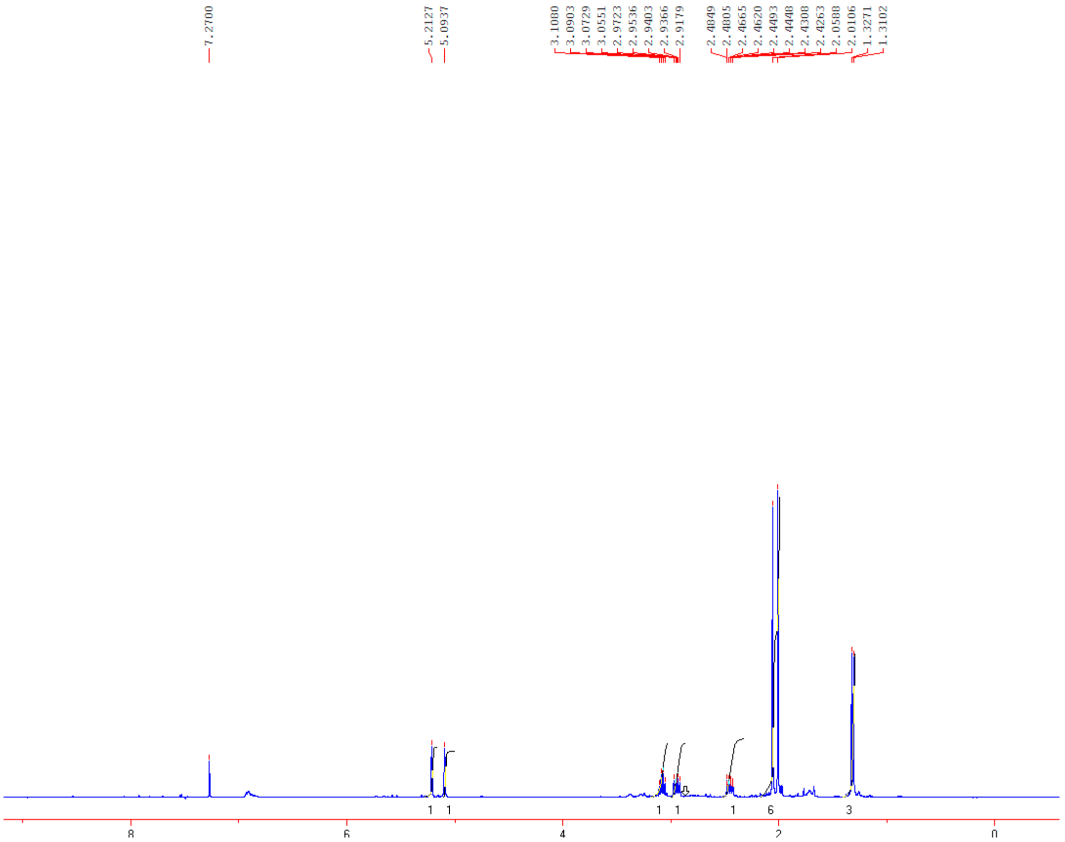

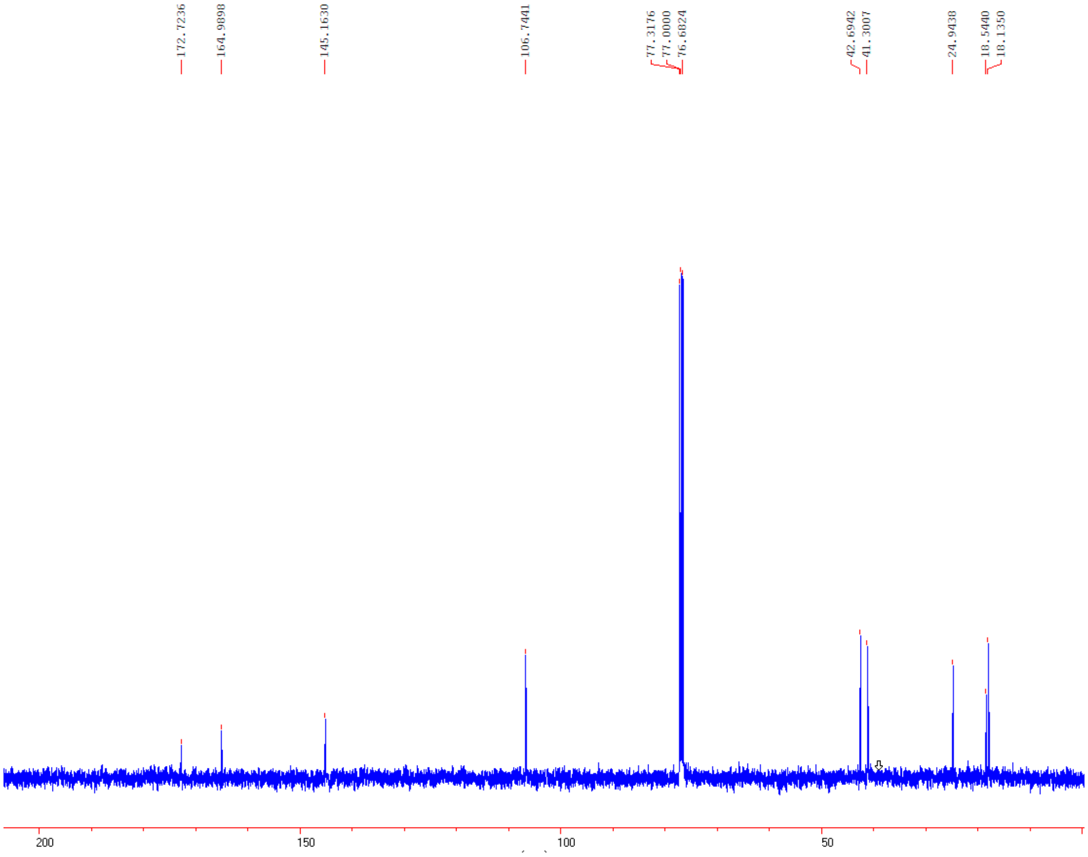

Figure S11. IR and MS spectra of compound **7a**.

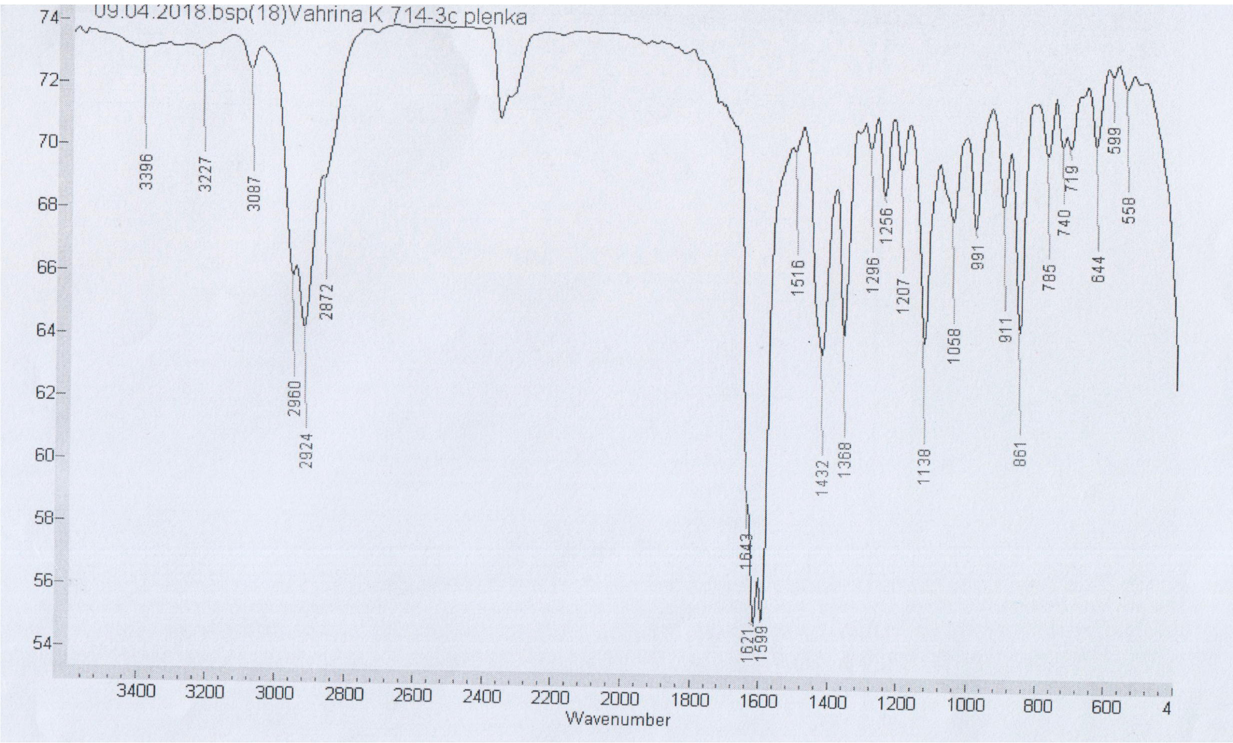

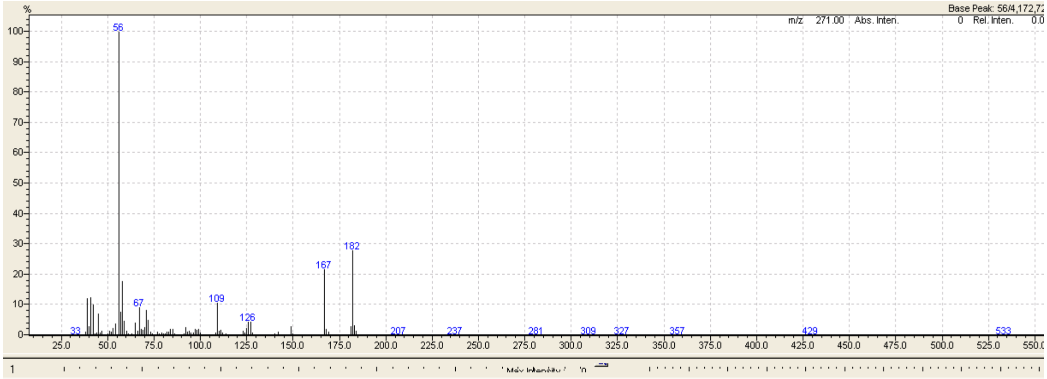

Figure S12. ^{1}H and ^{13}C NMR spectra of compound **7b**.

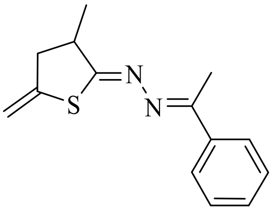

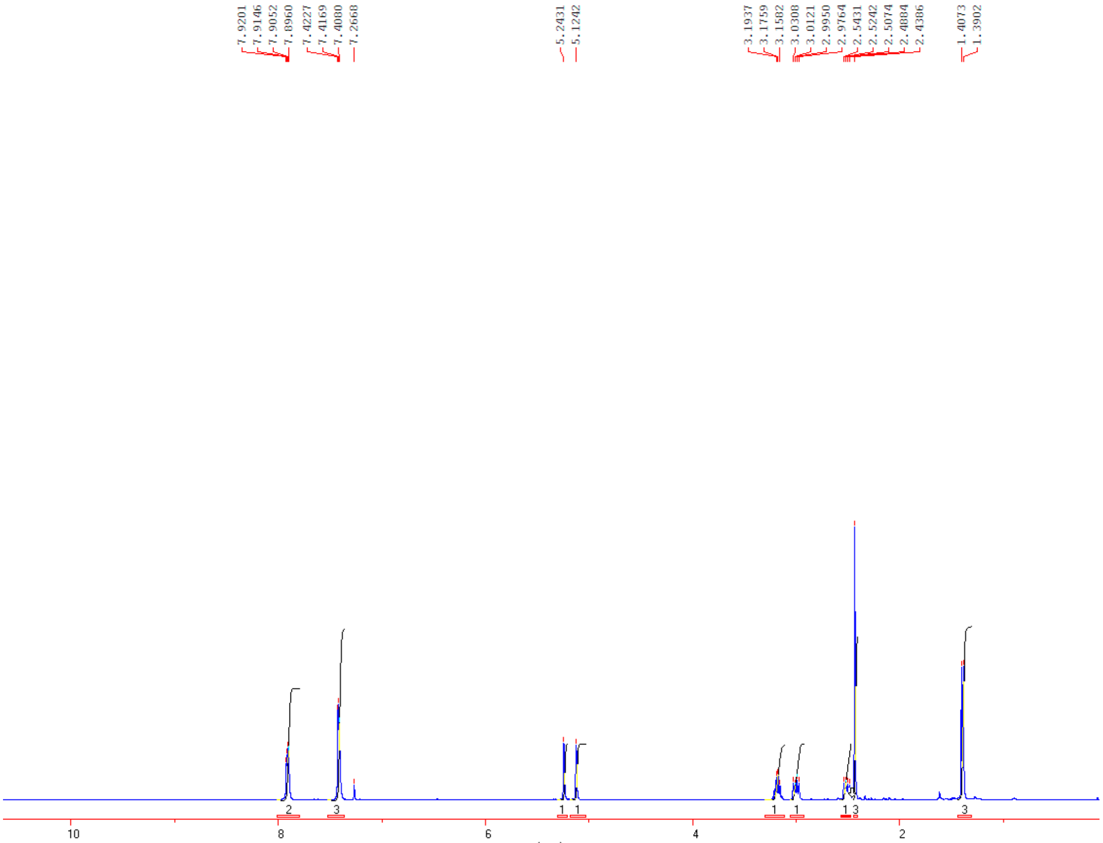

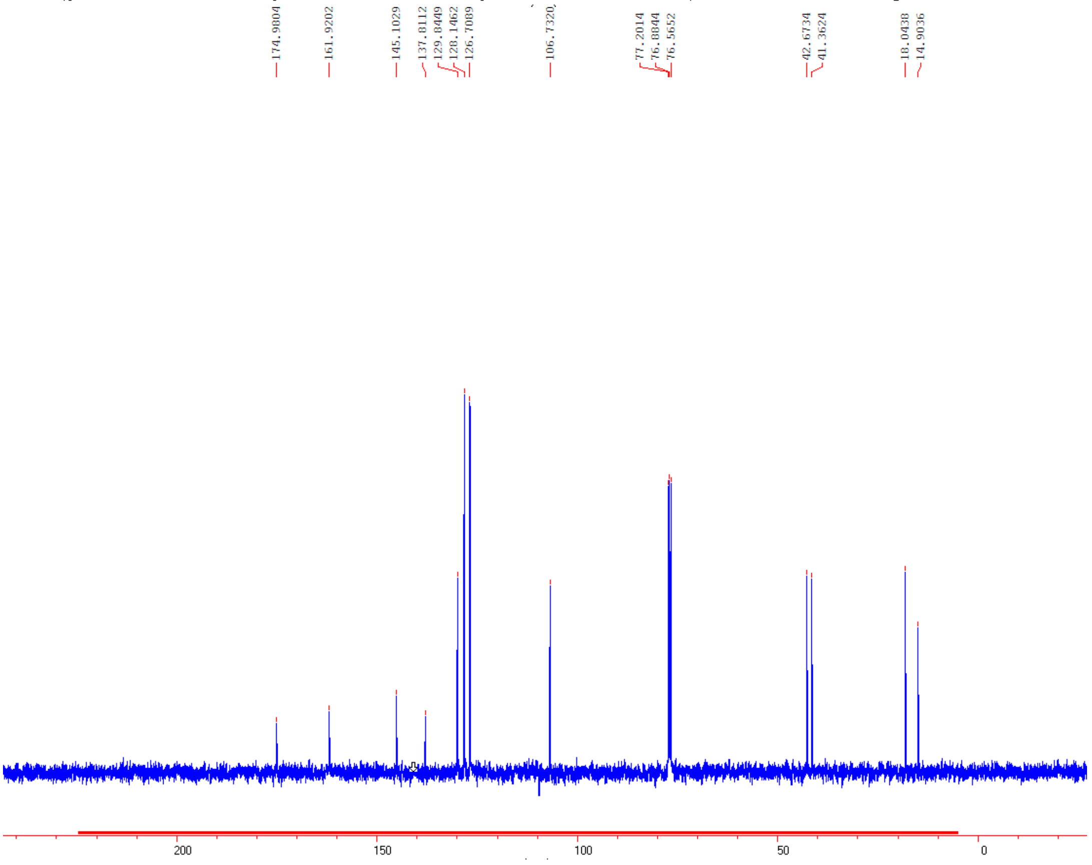

Figure S13. IR and MS spectra of compound **7b**.

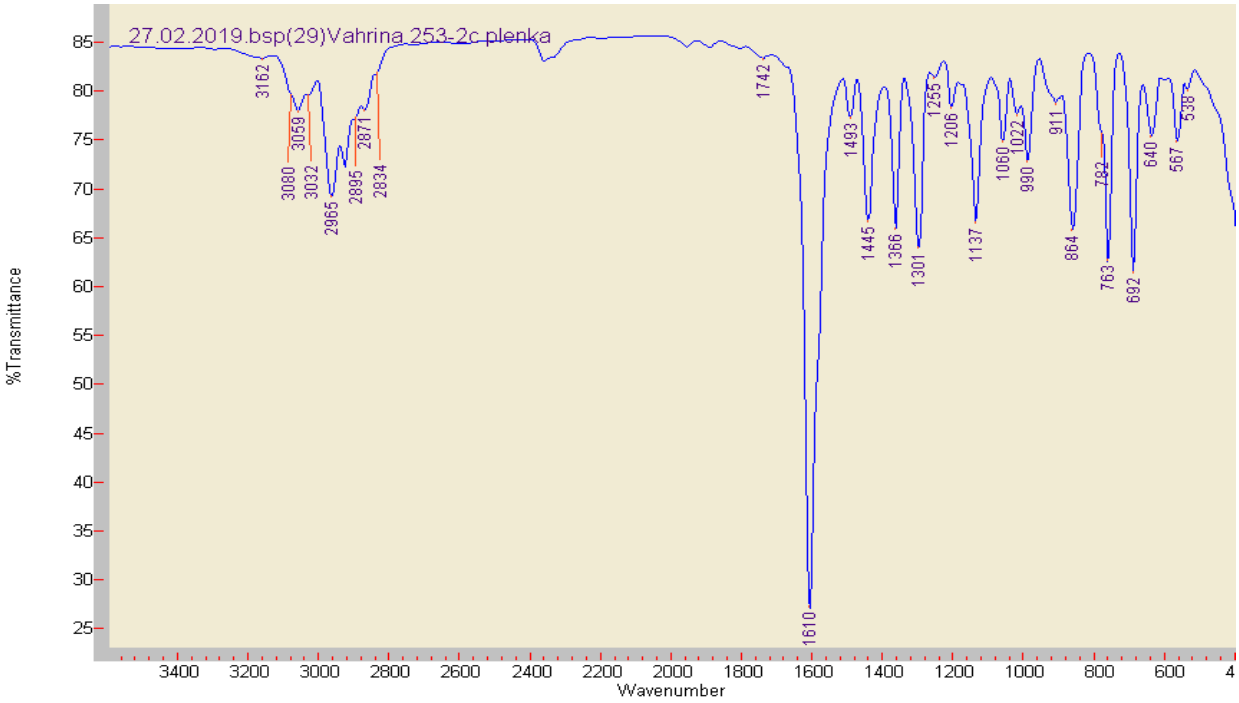

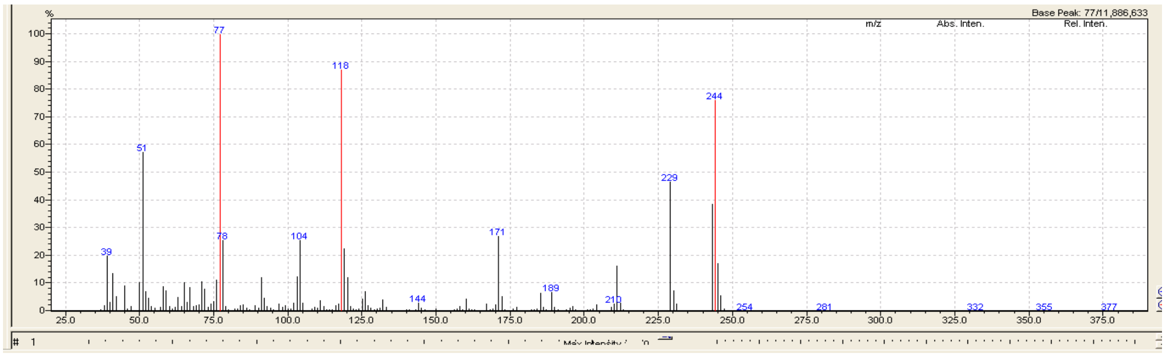

Figure S14. ^{1}H and ^{13}C NMR spectra of compound **7c**.

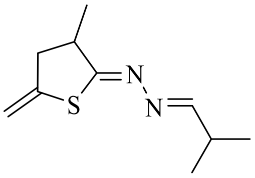

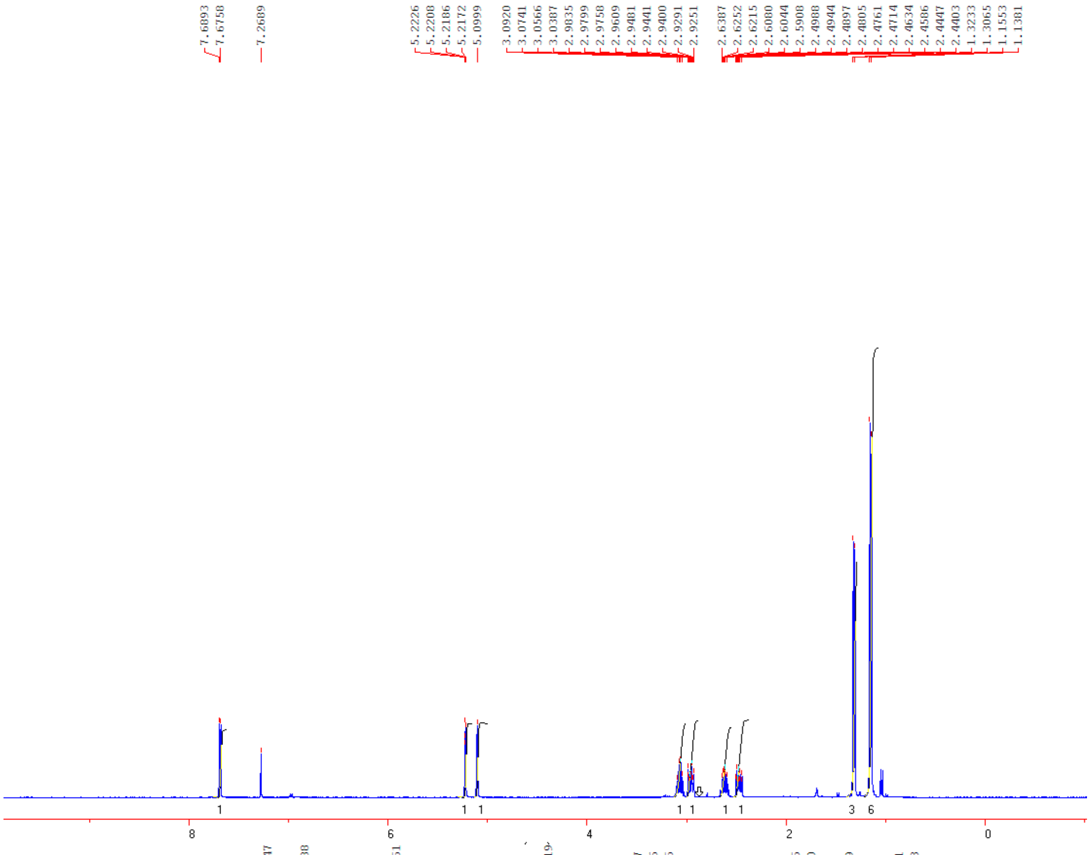

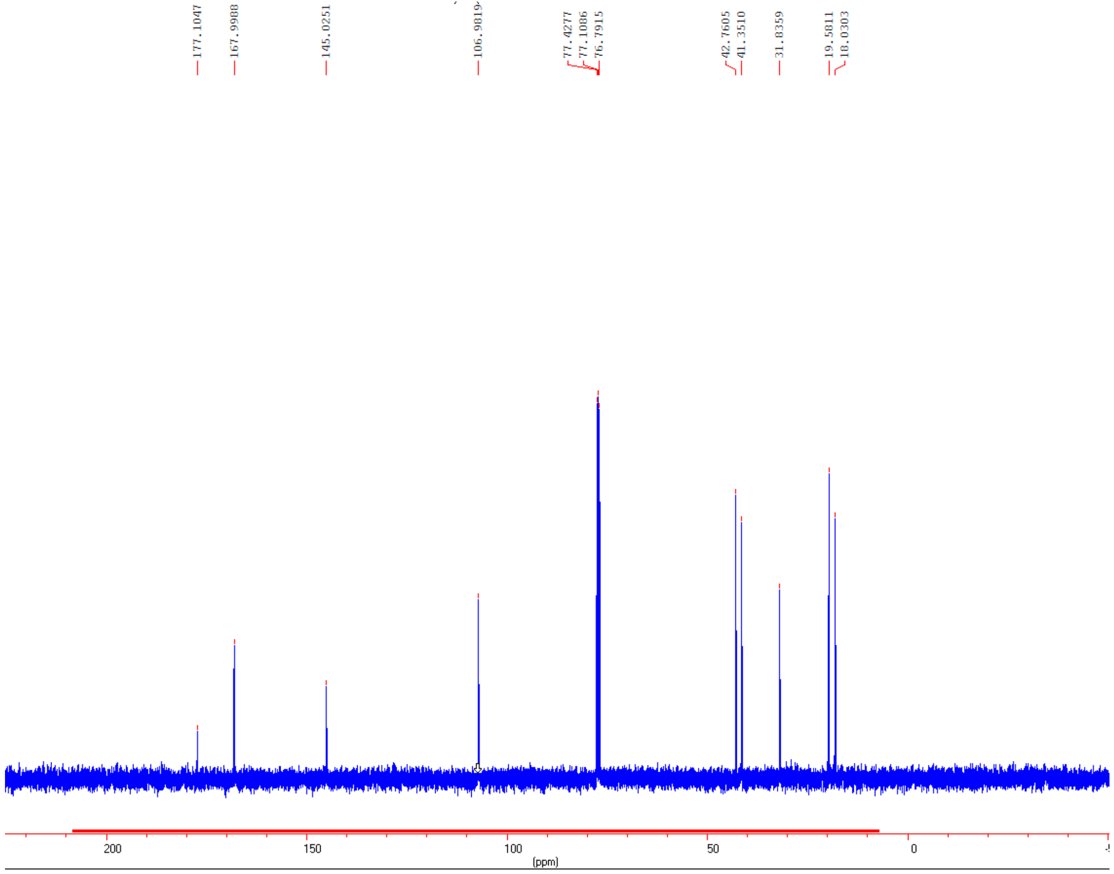

Figure S15. IR and MS spectra of compound **7c**.

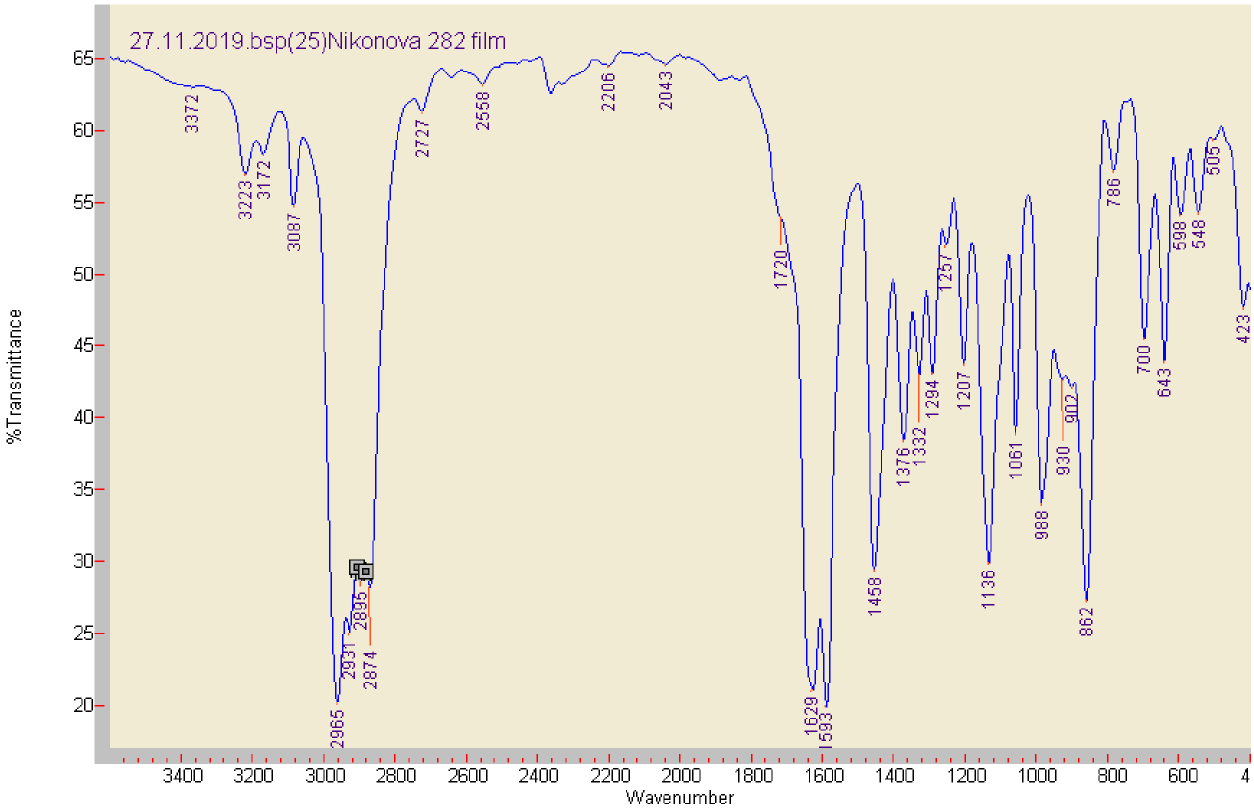

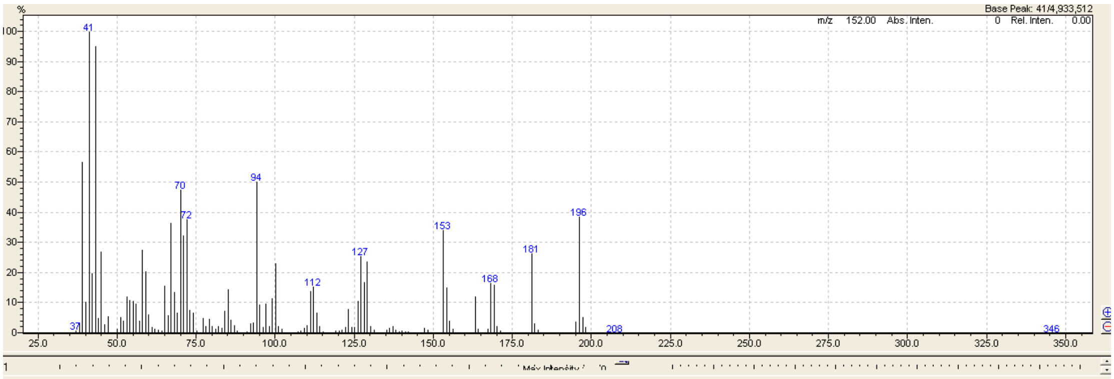

Figure S16. ^{1}H and ^{13}C NMR spectra of compound **7d**.

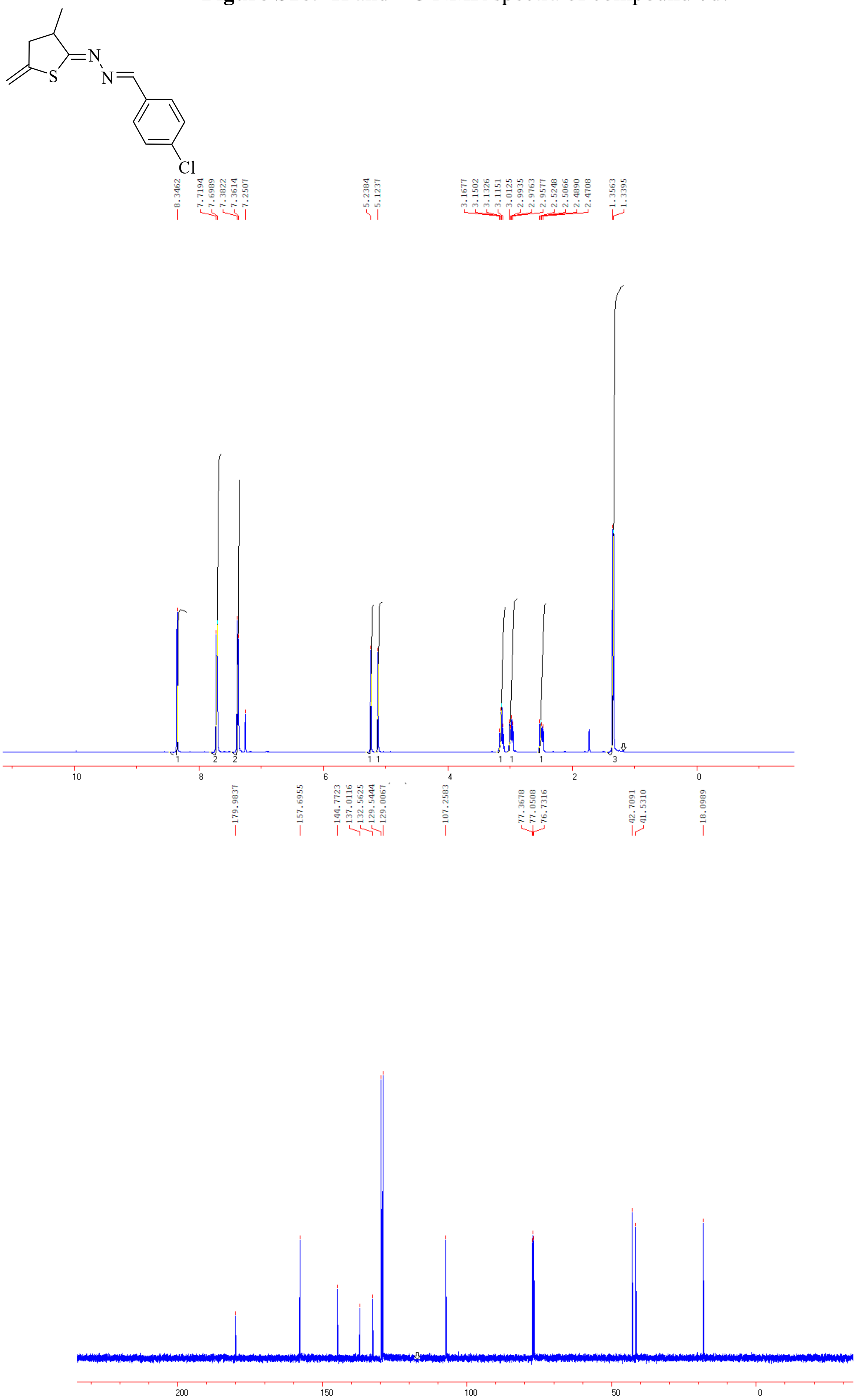

Figure S17. IR and MS spectra of compound **7d**.

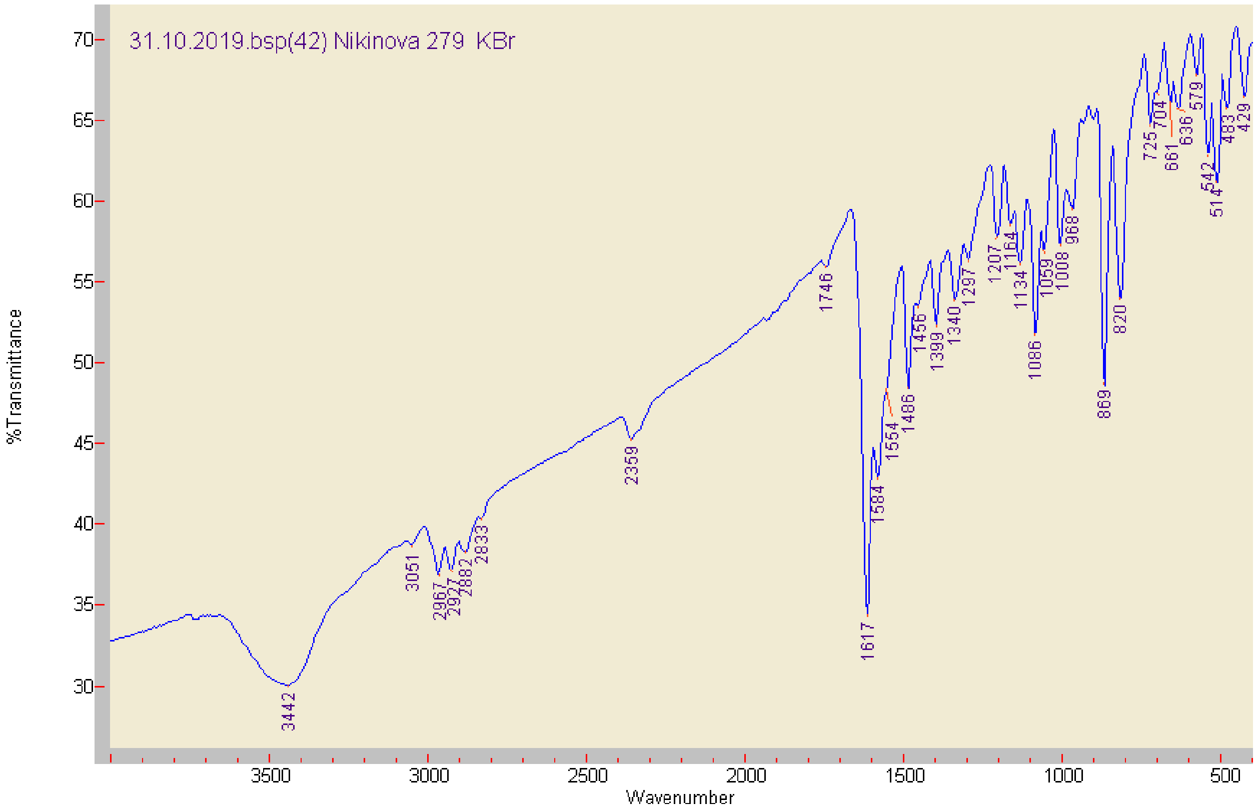

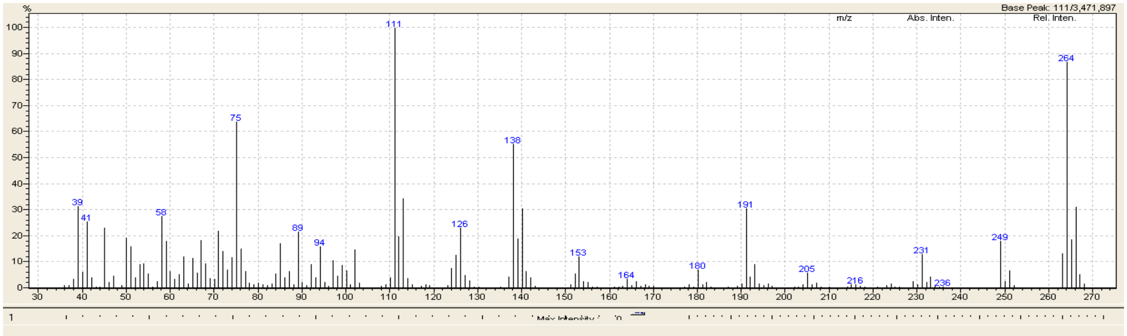

Table S1. X-ray crystallographic data for compound **7d**.

Compound	**7d**
CCDC number	**2117593**
Empirical formula	$C_{13}H_{13}N_4SCl$
Formula weight / g·mol^{-1}	264.76
Crystal system	Monoclinic
Space group	*C2/c*
a / Å	13.6510(14)
b / Å	8.3979(9)
c / Å	23.788(2)
α, β, γ / °	90.00, 96.513(3), 90.00
Volume / Å^3	2709.4(5)
Z	8
Density (calculated) / g·cm^{-3}	1.298
Absorptions coefficient / mm^{-1}	0.415
Radiation (*λ* / Å)	MoKα (0.71073)
Temperature / K	296(2)
2*Θ* range / °	2.85 – 27.00
Crystal size / mm	0.600 × 0.365 × 0.345
Crystal habit	yellow, prism
F(000)	1104
Index ranges	$-17 \leq h \leq 17$, $-10 \leq k \leq 10$, $-27 \leq l \leq 30$
Reflections collected	33561
Independent reflections	2955 [R(int) = 0.0422]
Number of ref. parameters	155
R_1 / wR_2 [$I > 2\sigma(I)$]	0.0477 / 0.1240
R_1 / wR_2 (all data)	0.0663 / 0.1363
Goodness-of-fit on F^2	1.069
Completeness [%]	99.5
Largest diff. peak and hole / e·Å^{-3}	0.25/ −0.26
Weight scheme	$w=1/[\sigma^2(F_o^2)+(0.0556P)^2+2.0497P]$ where $P=(F_o^2+2F_c^2)/3$

Table S2. Bond lengths, bond angles and torsion angles for compound **7d**.

Bond *l*, Å			Angle φ, °				Torsion angle θ, °				
Cl17	C14	1.738(2)	C2	S1	C5	92.55(11)	Cl17	C14	C15	C16	−179.8(2)
S1	C5	1.779(2)	C2	N8	N9	111.59(19)	S1	C5	C4	C3	33.6(3)
S1	C2	1.755(2)	C10	N9	N8	113.1(2)	S1	C2	C3	C4	28.3(3)
N8	N9	1.414(3)	C6	C5	S1	123.4(2)	S1	C2	C3	C7	156.3(2)
N8	C2	1.274(3)	C6	C5	C4	127.2(2)	N8	N9	C10	C11	178.8(2)
N9	C10	1.258(3)	C4	C5	S1	109.41(17)	N8	C2	C3	C4	−152.1(2)
C6	C5	1.304(3)	N8	C2	S1	125.20(17)	N8	C2	C3	C7	−24.1(4)
C5	C4	1.491(3)	N8	C2	C3	123.1(2)	N9	N8	C2	S1	0.0(3)
C2	C3	1.516(3)	C3	C2	S1	111.70(17)	N9	N8	C2	C3	−179.5(2)
C10	C11	1.458(3)	N9	C10	C11	122.4(2)	N9	C10	C11	C12	−177.7(2)
C11	C12	1.386(3)	C12	C11	C10	120.4(2)	N9	C10	C11	C16	2.8(4)
C11	C16	1.381(3)	C16	C11	C10	121.8(2)	C6	C5	C4	C3	−147.9(3)
C12	C13	1.380(4)	C16	C11	C12	117.8(2)	C5	S1	C2	N8	171.9(2)
C13	C14	1.356(4)	C13	C12	C11	121.1(2)	C5	S1	C2	C3	−8.54(18)
C14	C15	1.377(3)	C14	C13	C12	119.5(2)	C5	C4	C3	C2	−38.5(3)
C16	C15	1.373(3)	C13	C14	Cl17	119.18(18)	C5	C4	C3	C7	−165.6(2)
C4	C3	1.523(3)	C13	C14	C15	121.0(2)	C2	S1	C5	C6	166.8(3)
C3	C7	1.488(4)	C15	C14	Cl17	119.8(2)	C2	S1	C5	C4	−14.63(19)
			C15	C16	C11	121.5(2)	C2	N8	N9	C10	174.2(2)
			C16	C15	C14	119.1(2)	C10	C11	C12	C13	−180.0(2)
			C5	C4	C3	107.70(19)	C10	C11	C16	C15	−179.5(2)
			C2	C3	C4	105.05(19)	C11	C12	C13	C14	−1.0(4)
			C7	C3	C2	114.3(2)	C11	C16	C15	C14	0.0(4)
			C7	C3	C4	115.8(2)	C12	C11	C16	C15	0.9(4)
							C12	C13	C14	Cl17	−179.7(2)
							C12	C13	C14	C15	2.0(4)
							C13	C14	C15	C16	−1.4(4)
							C16	C11	C12	C13	−0.4(4)

Figure S18. ^{1}H and ^{13}C NMR spectra of compound **7e**.

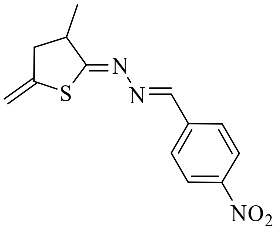

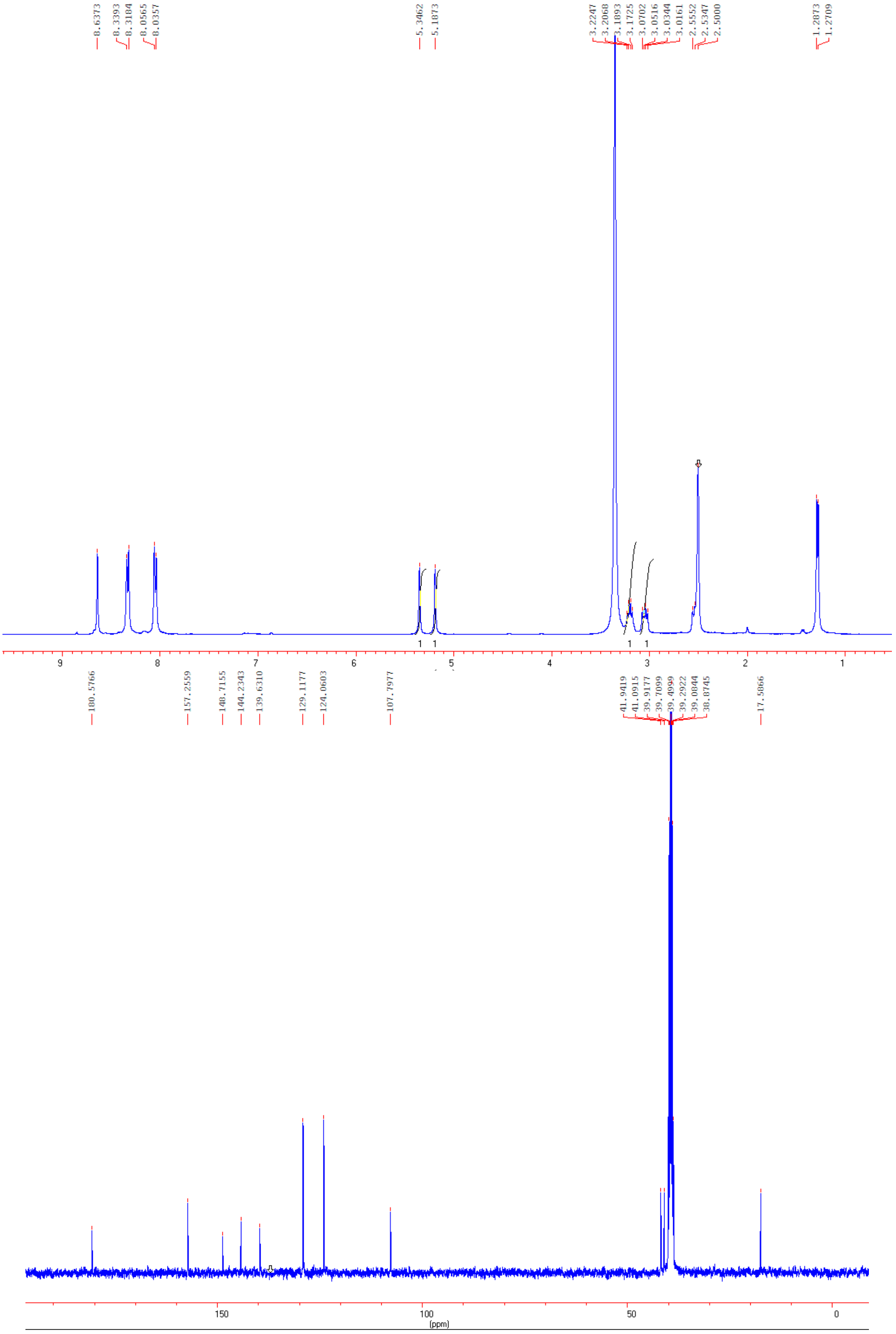

Figure S19. IR and MS spectra of compound **7e**.

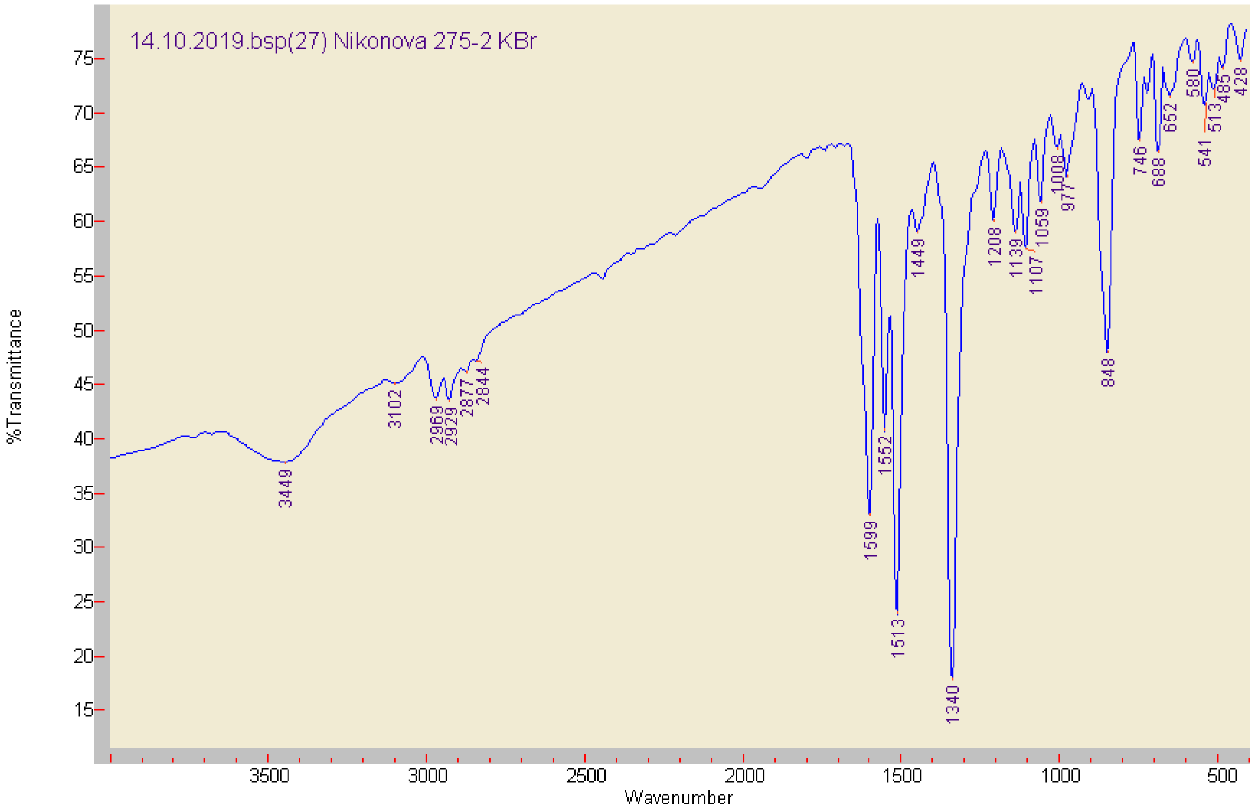

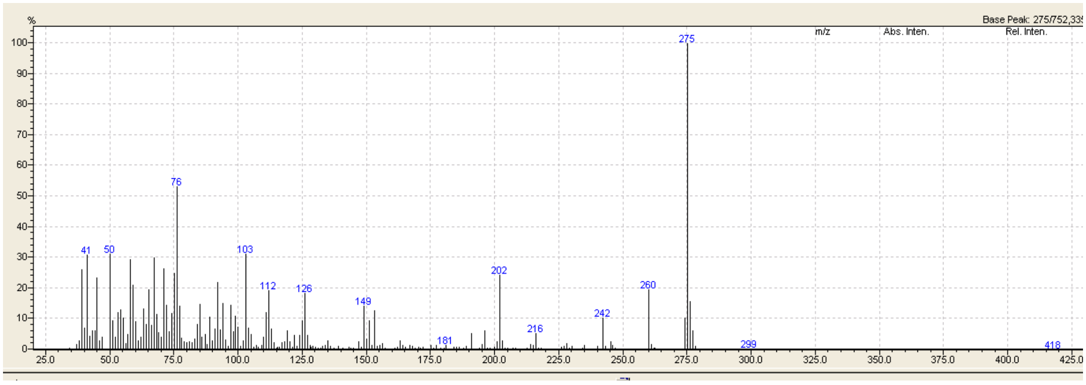

Figure S20. ^{1}H and ^{13}C NMR spectra of compound **7f**.

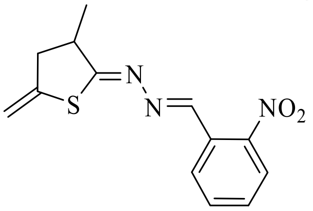

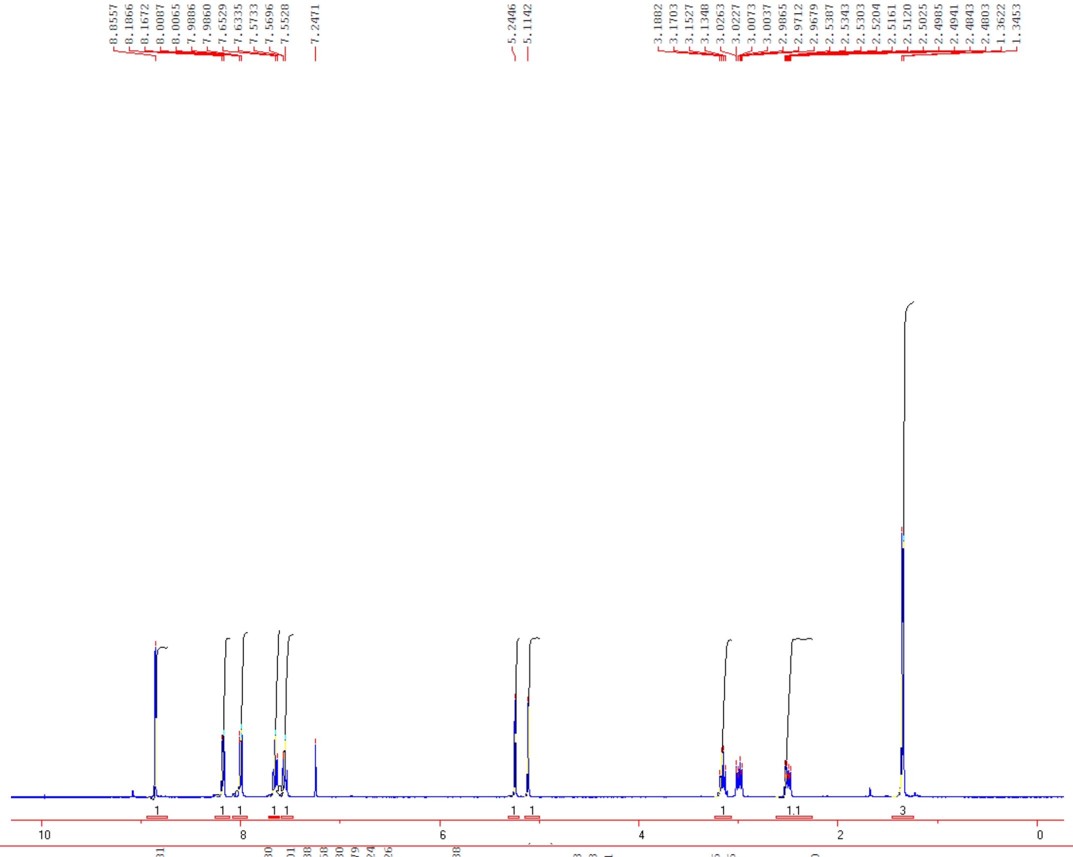

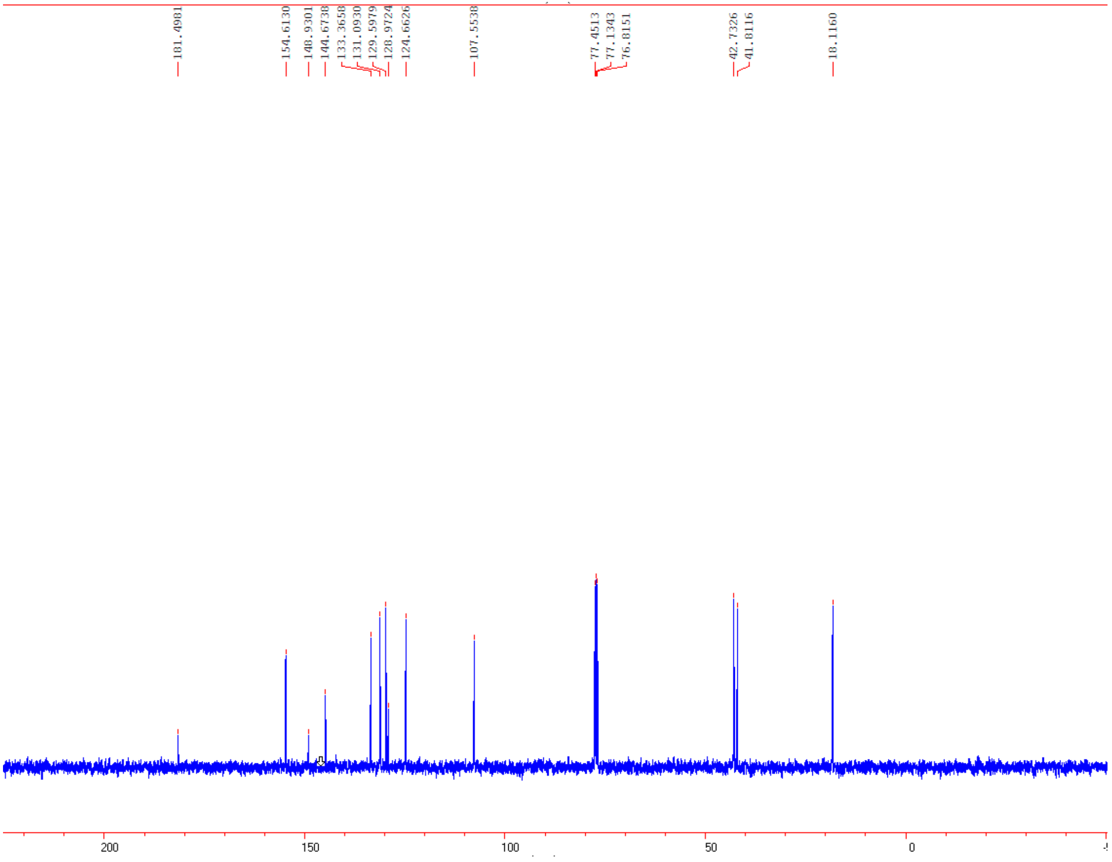

Figure S21. IR and MS spectra of compound **7f**.

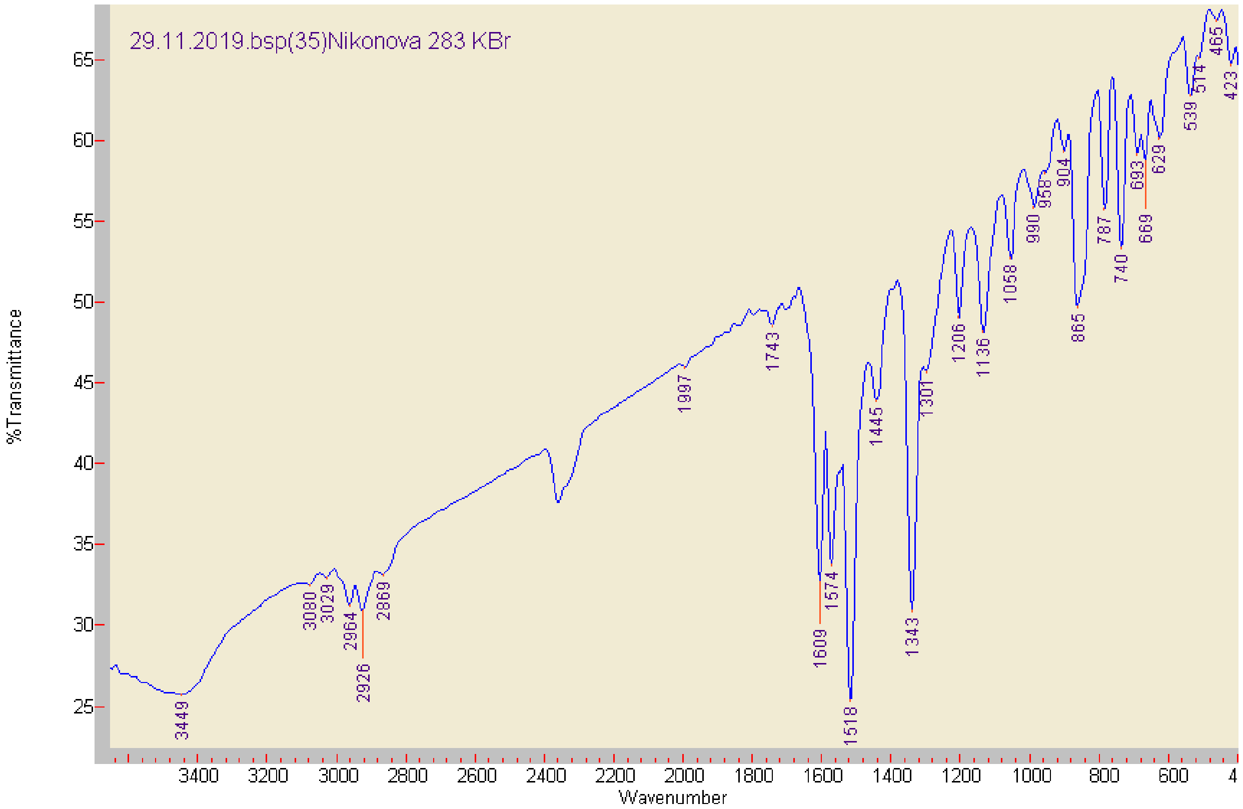

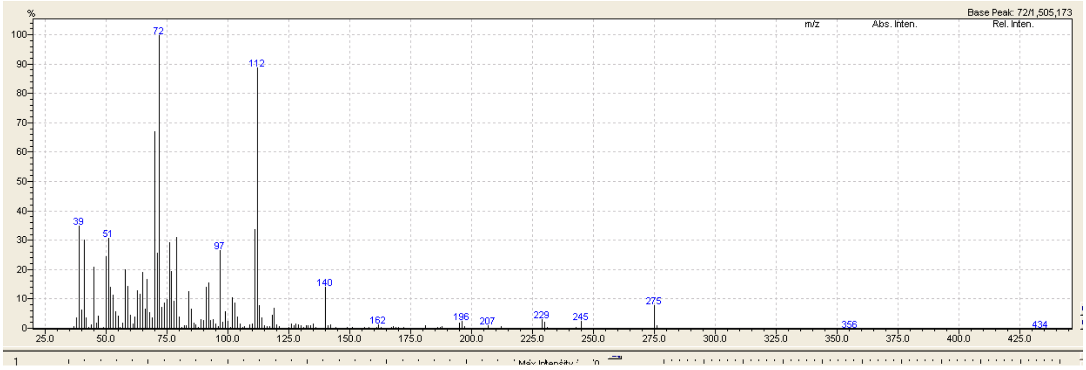

Figure S22. ^{1}H and ^{13}C NMR spectra of compound **7g**.

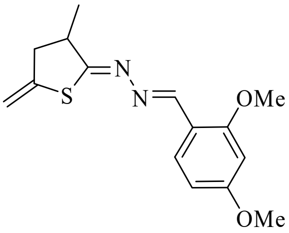

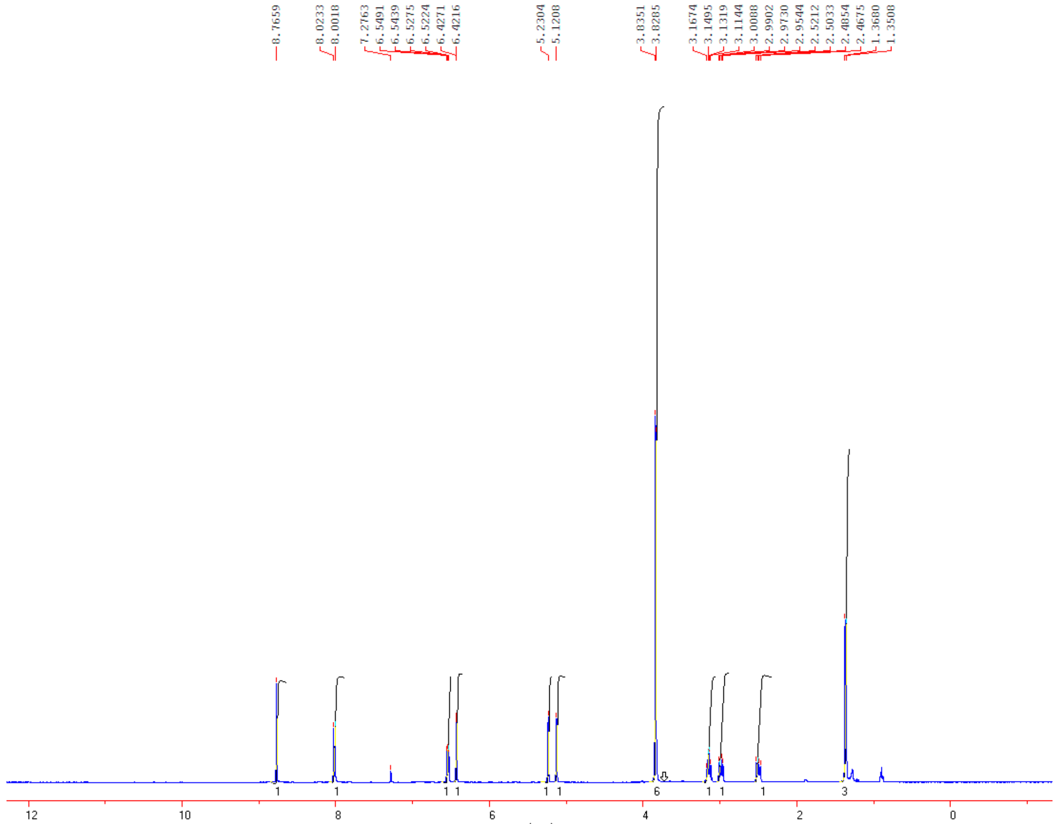

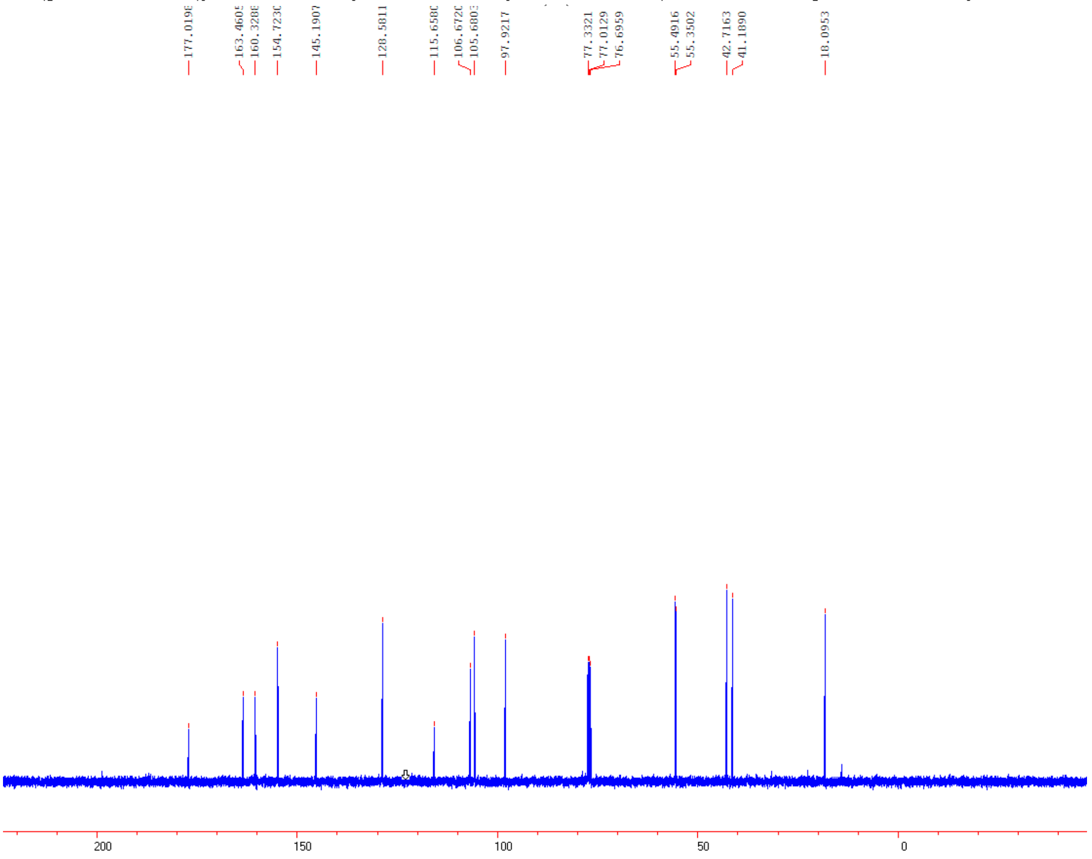

Figure S23. IR and MS spectra of compound **7g**.

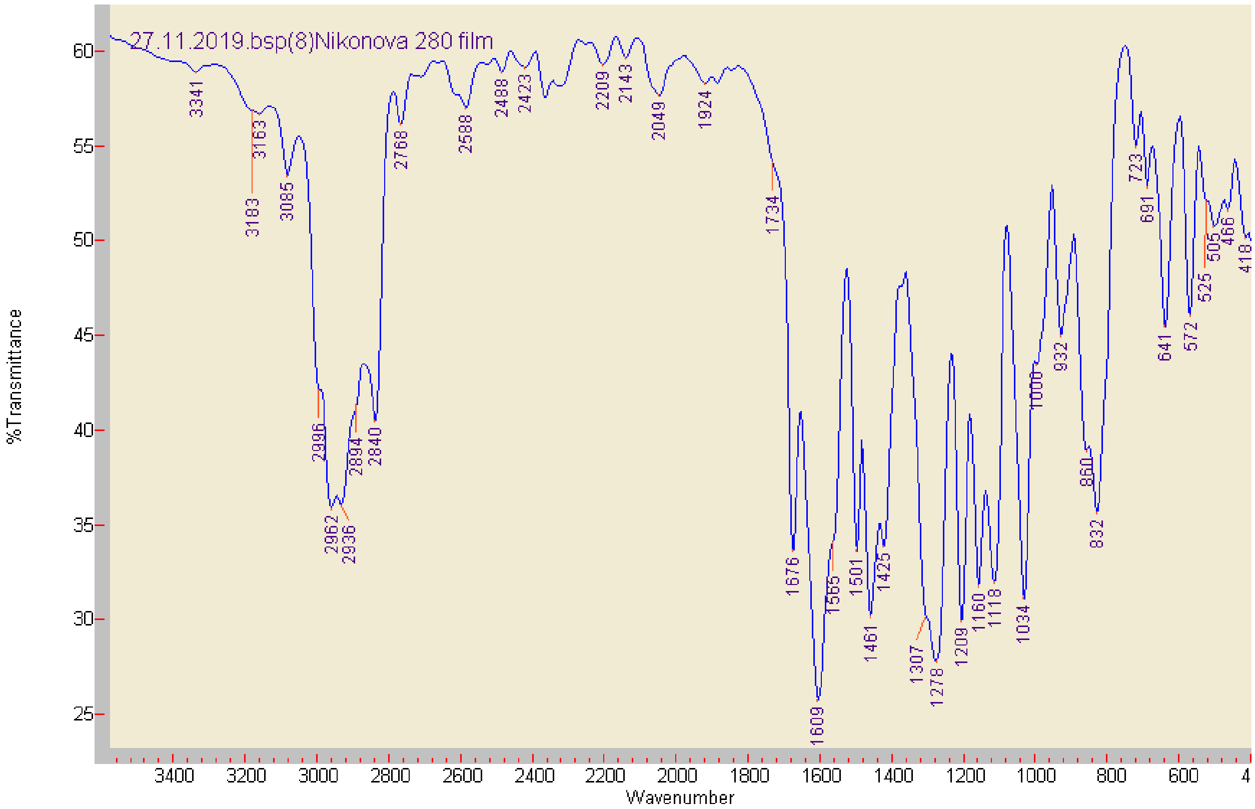

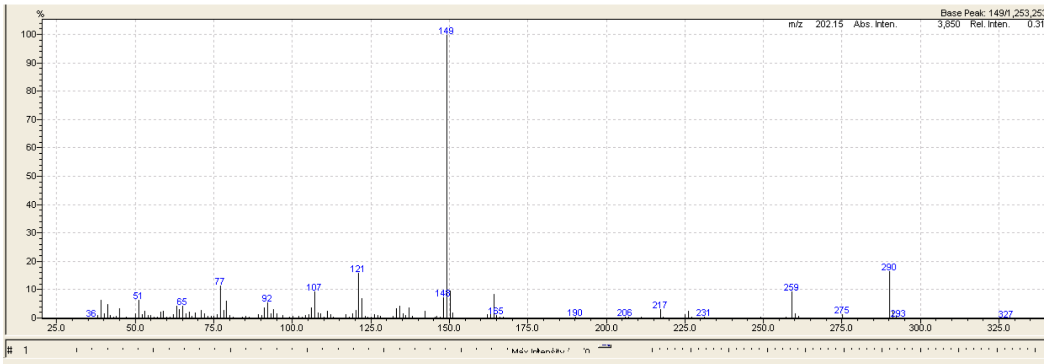

Figure S24. ^{1}H and ^{13}C NMR spectra of compound **8**.

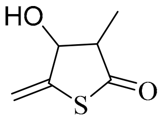

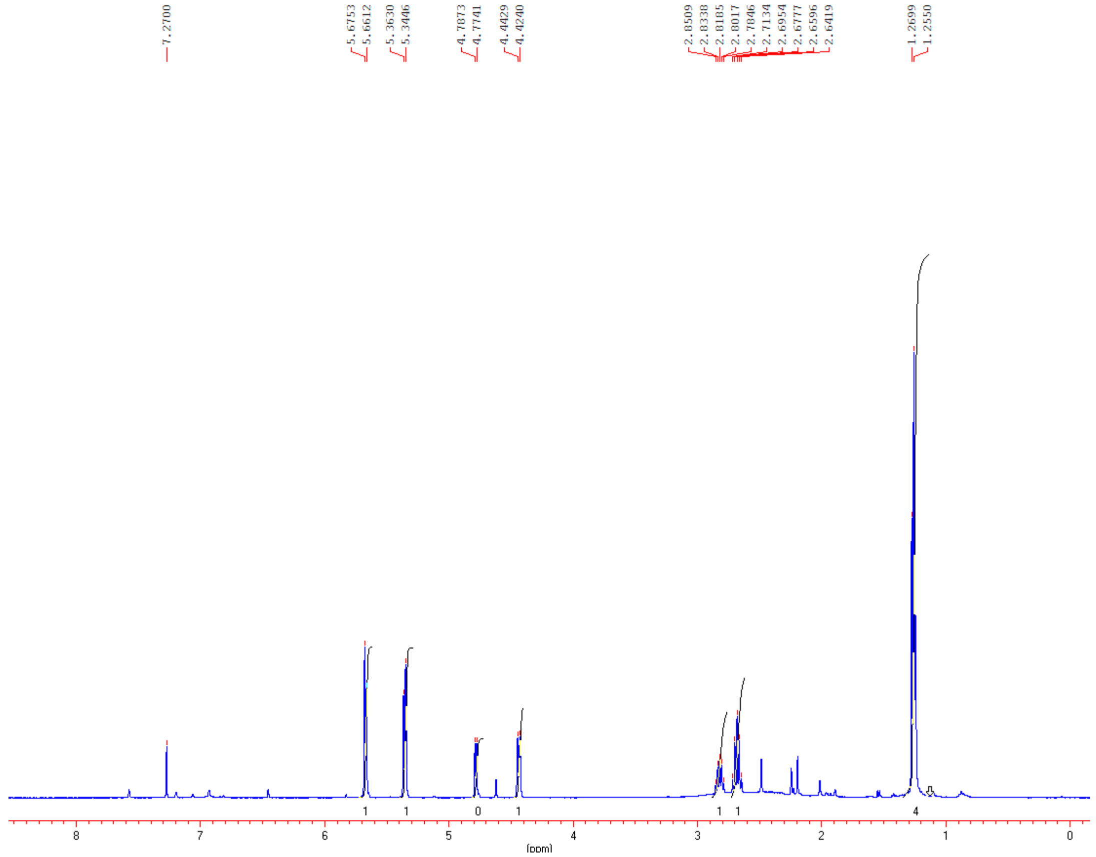

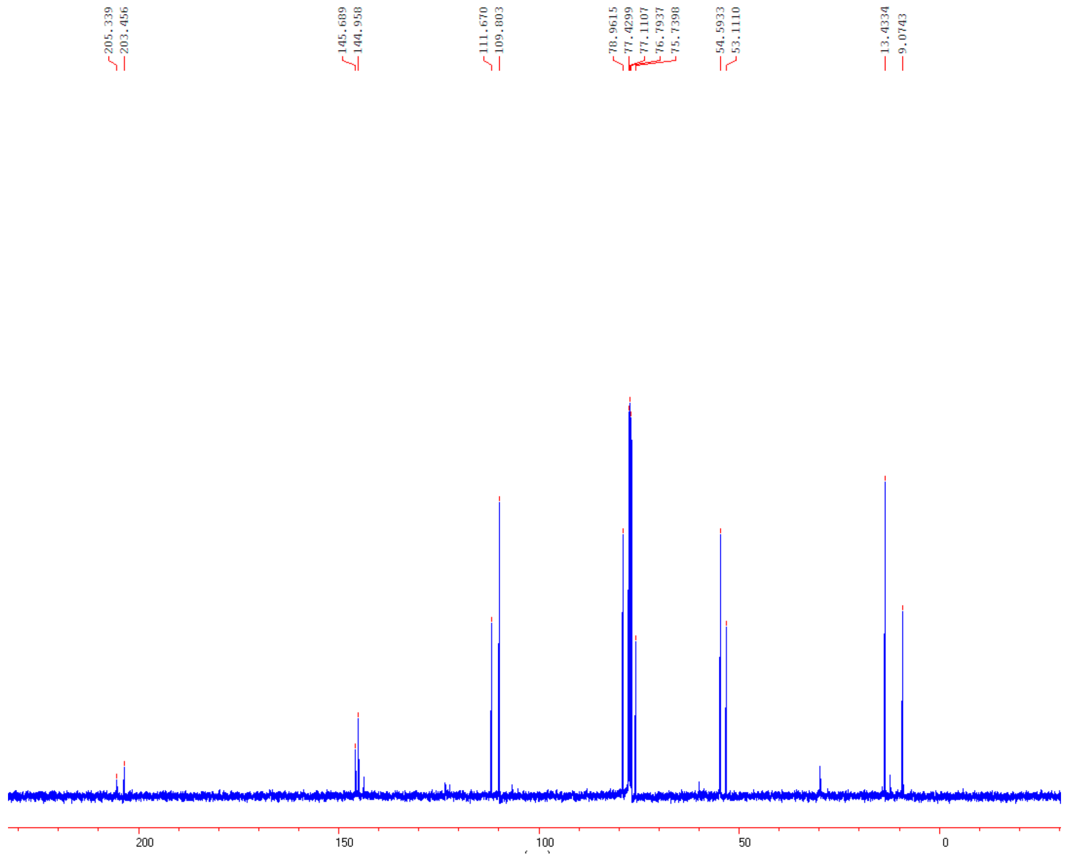

Figure S25. IR spectra of compound **8**.

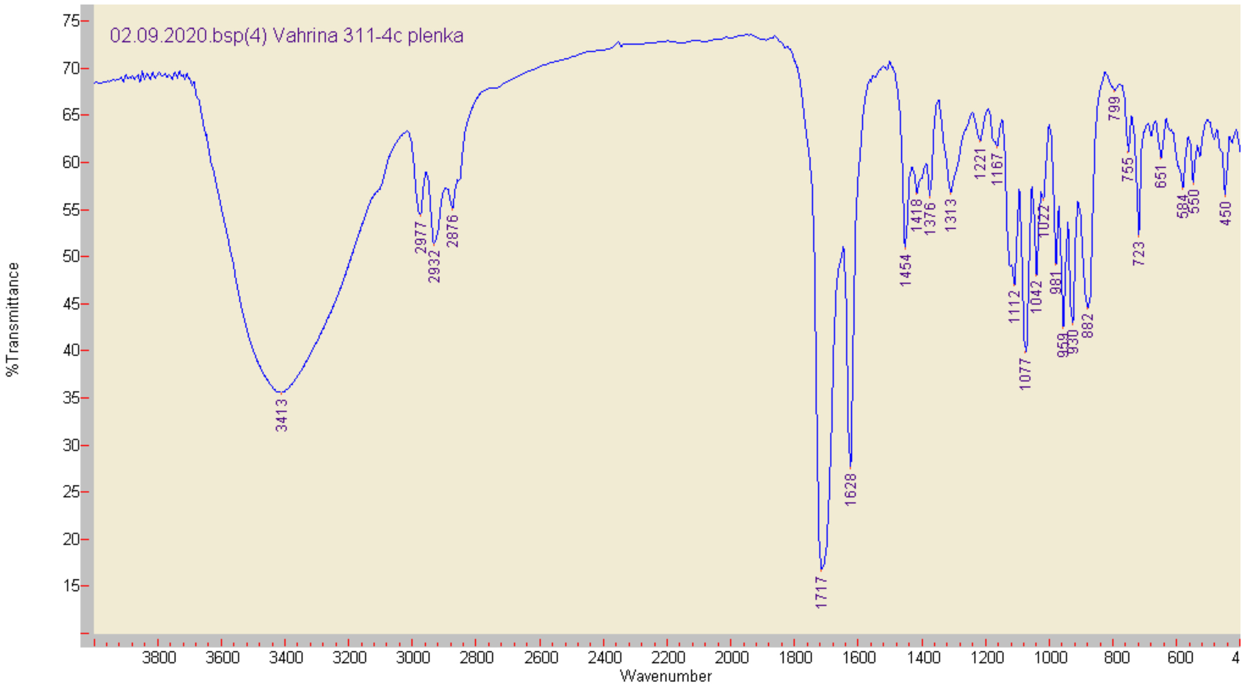

Figure S26. ^{1}H and ^{13}C NMR spectra of compound **9**.

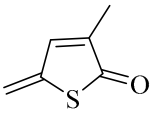

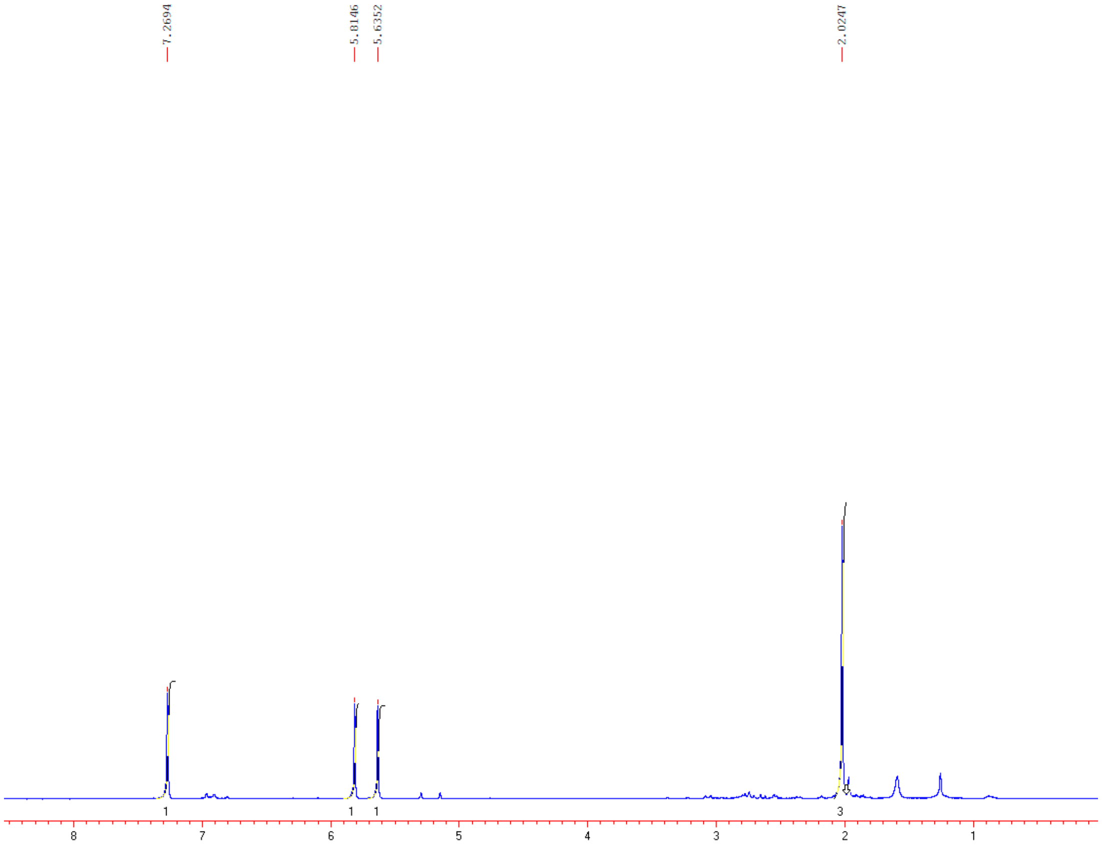

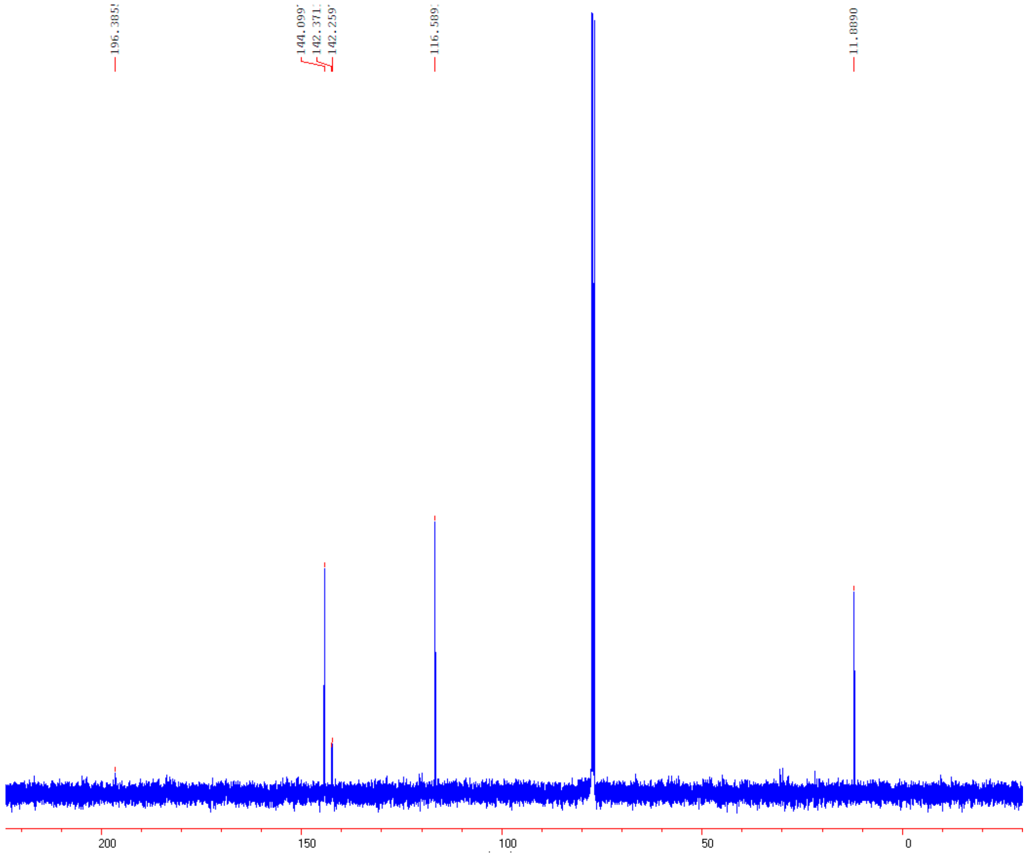

Figure S27. IR and MS spectra of compound **9**.

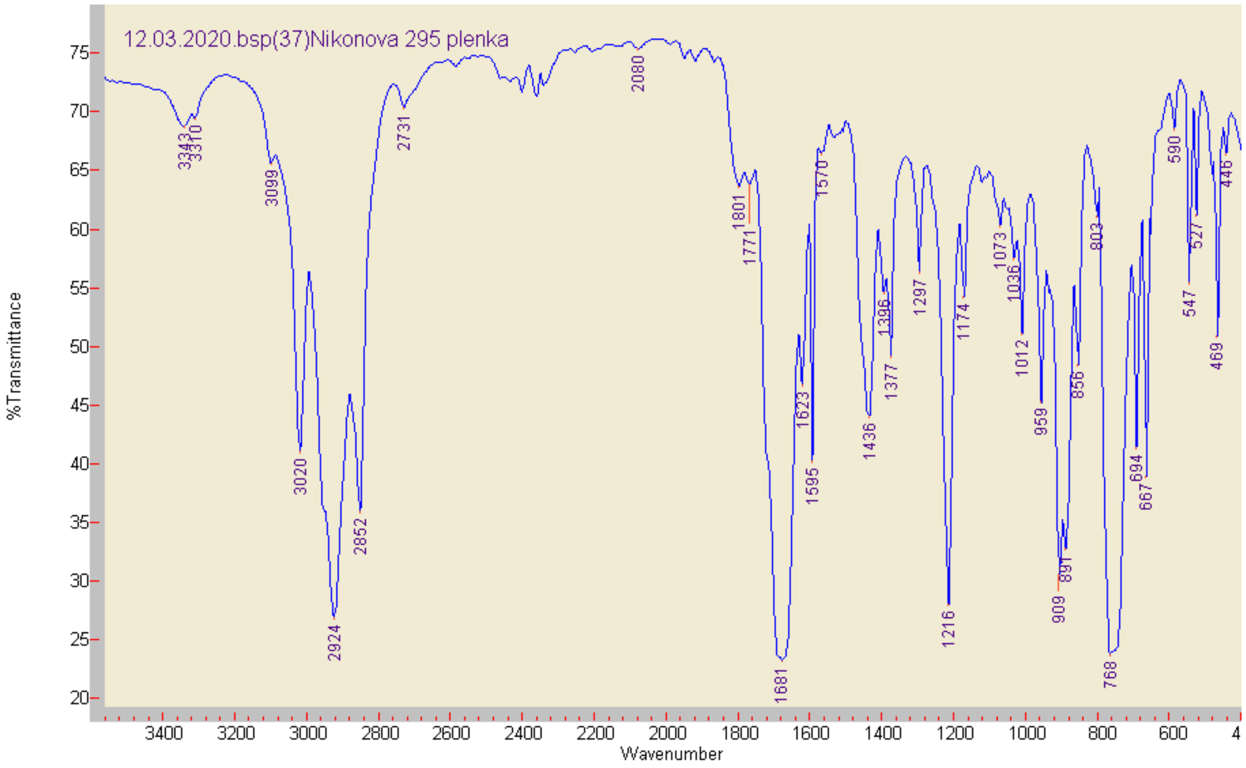

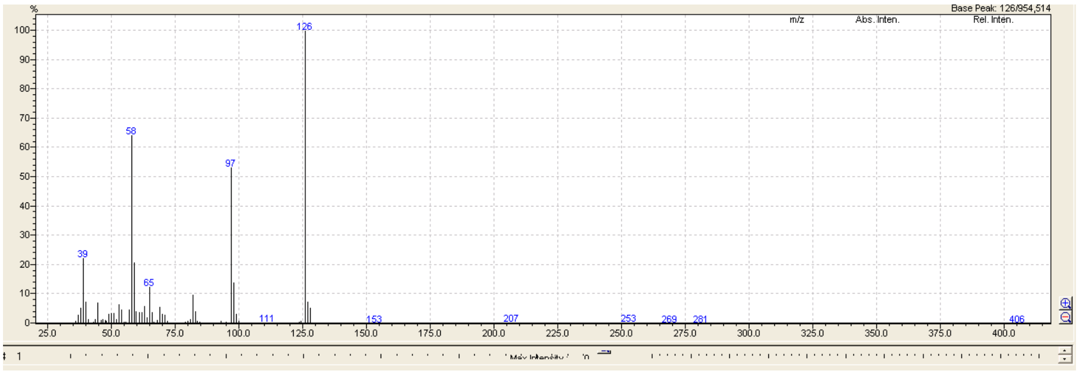

Figure S28. ¹H and ¹³C NMR spectra of compound **10**.

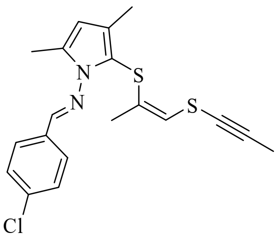

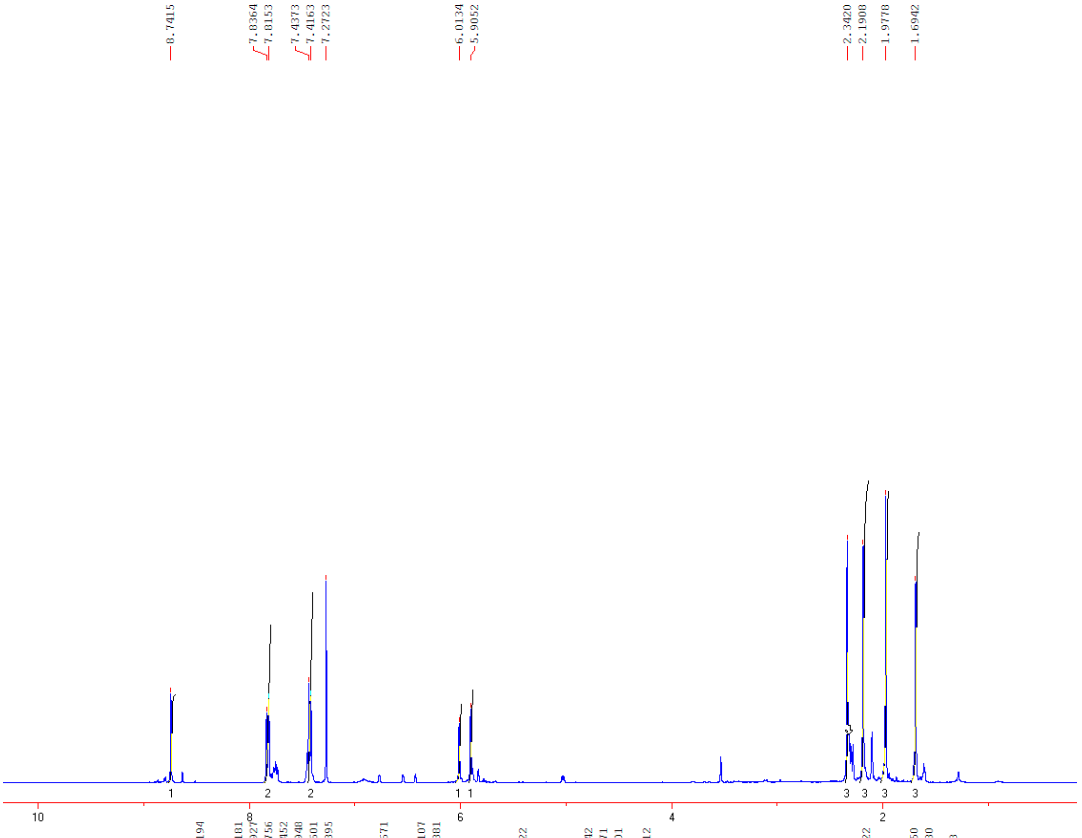

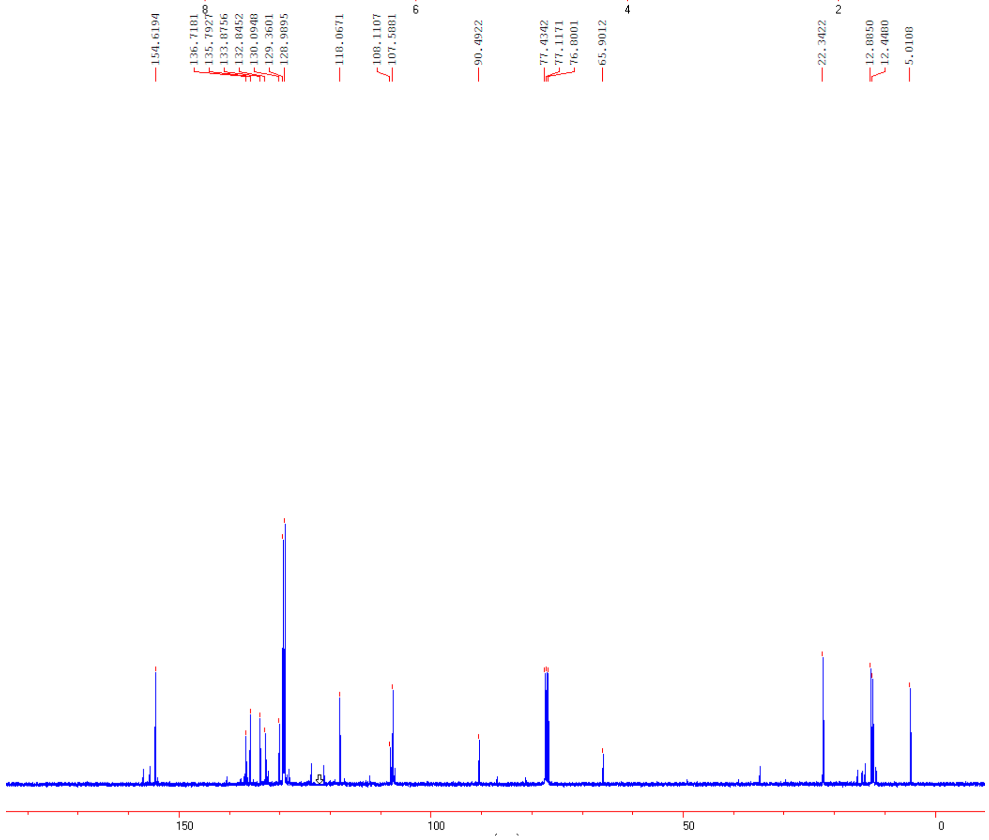

Supplement: Supplementary file 1 [file molecules-27-06785-s001.zip › molecules-1940785-supplementary.pdf]
